# Intracellular Fate of the Photosensitizer Chlorin e4 with Different Carriers and Induced Metabolic Changes Studied by ^1^H NMR Spectroscopy

**DOI:** 10.3390/pharmaceutics15092324

**Published:** 2023-09-15

**Authors:** Martina Vermathen, Tobias Kämpfer, Jean-Marc Nuoffer, Peter Vermathen

**Affiliations:** 1 Department of Chemistry, Biochemistry and Pharmaceutical Sciences, University of Bern, 3012 Bern, Switzerland; tobias.kaempfer@unibe.ch; 2 Department of BioMedical Research, University of Bern, 3008 Bern, Switzerland; 3 Institute of Clinical Chemistry, Bern University Hospital, 3010 Bern, Switzerland; jean-marc.nuoffer@insel.ch; 4 Department of Pediatric Endocrinology, Diabetology and Metabolism, University Children’s Hospital of Bern, 3010 Bern, Switzerland; 5 University Institute of Diagnostic and Interventional Neuroradiology, Bern University Hospital, University of Bern, 3010 Bern, Switzerland; 6 Translational Imaging Center (TIC), Swiss Institute for Translational and Entrepreneurial Medicine, 3010 Bern, Switzerland

**Keywords:** photodynamic therapy, photosensitizer, porphyrinic compounds, polymeric carriers, nanoparticles, metabolic profile, high-resolution magic angle spinning NMR, diffusion-ordered spectroscopy, polyvinylpyrrolidone, Kolliphor

## Abstract

Porphyrinic photosensitizers (PSs) and their nano-sized polymer-based carrier systems are required to exhibit low dark toxicity, avoid side effects, and ensure high in vivo tolerability. Yet, little is known about the intracellular fate of PSs during the dark incubation period and how it is affected by nanoparticles. In a systematic study, high-resolution magic angle spinning NMR spectroscopy combined with statistical analyses was used to study the metabolic profile of cultured HeLa cells treated with different concentrations of PS chlorin e4 (Ce4) alone or encapsulated in carrier systems. For the latter, either polyvinylpyrrolidone (PVP) or the micelle-forming polyethylene glycol (PEG)-polypropylene glycol triblock copolymer Kolliphor P188 (KP) were used. Diffusion-edited spectra indicated Ce4 membrane localization evidenced by Ce4 concentration-dependent chemical shift perturbation of the cellular phospholipid choline resonance. The effect was also visible in the presence of KP and PVP but less pronounced. The appearance of the PEG resonance in the cell spectra pointed towards cell internalization of KP, whereas no conclusion could be drawn for PVP that remained NMR-invisible. Multivariate statistical analyses of the cell spectra (PCA, PLS-DA, and oPLS) revealed a concentration-dependent metabolic response upon exposure to Ce4 that was attenuated by KP and even more by PVP. Significant Ce4-concentration-dependent alterations were mainly found for metabolites involved in the tricarboxylic acid cycle and the phosphatidylcholine metabolism. The data underline the important protective role of the polymeric carriers following cell internalization. Moreover, to our knowledge, for the first time, the current study allowed us to trace intracellular PS localization on an atomic level by NMR methods.

## 1. Introduction

**Figure 1:**
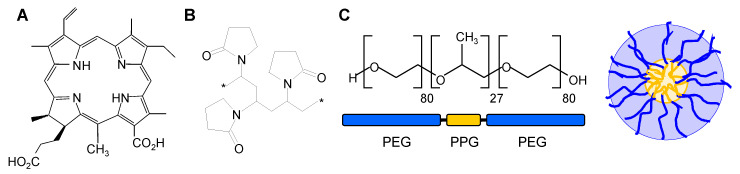
(**A**) Structure of the photosensitizer chlorin e4 (Ce4); (**B**) building block of the polymeric network formed by polyvinylpyrrolidone (PVP, MW_av_ 10 kDa), *: repeated building blocks [ ]_n_; (**C**) polyethylene glycol (PEG)—polypropylene glycol (PPG) triblock copolymer PEG-PPG-PEG Kolliphor P 188 (KP) and a sketch of a KP-micelle.

The chlorophyll-based porphyrinic derivatives of chlorin e6 (Ce6) are efficient photosensitizers (PSs) in photodynamic therapy (PDT) of cancer and non-cancerous diseases. PDT relies on the activation of PS by light irradiation, upon which the excited PS transfers energy to molecular oxygen, which leads to the formation of cytotoxic singlet oxygen and reactive oxygen species (ROS) in its immediate surroundings [1]. Among the favorable properties of Ce6 derivatives is their intense light absorption in the wavelength region of 600–700 nm [2,3], where tissue penetration of light is deeper than at shorter wavelengths [4]. Several Ce6 derivatives have received approval for specific applications of cancer [2,5], and the sodium salt of chlorin e4 (Ce4, Figure 1A) has been recently patented for use in PDT of different cancers, including ovarian cancer under the trademark Photosoft [6,7].

With the development of so-called third-generation PSs [5], the use of nano-platforms for drug delivery has become an essential part of PDT to improve PS efficiency [8]. Nanoparticle (NP)-based carriers can improve the solubility and bioavailability of the often lipophilic and aggregating porphyrinic PSs [8]. Further, NP surface modifications like polyethylene glycol (PEG) can improve PS stability during transport in the blood by reducing PS removal through macrophages or the reticuloendothelial system [9,10]. Based on their large size, NPs can accumulate in tumor tissue via the enhanced permeability and retention (EPR) effect of the tumor vasculature that is more permeable for macromolecules than normal tissue [11]. Finally, the NP surface offers possibilities for chemical modifications aiming at specific targeting [12]. Cell entry of NPs normally takes place via endocytic pathways, where the specific route is determined by the cell type and by the nature of the NP [13]. Physicochemical properties of NPs, like size, shape, chemical composition, and surface charge, determine cell uptake and intracellular trafficking [13,14,15,16]. Among the various materials applied for nano-sized delivery systems, polyvinylpyrrolidone (PVP, Figure 1B) [17,18,19] and block copolymer micelles (BCMs, Figure 1C) [20,21,22] consisting of polymers with PEG and polypropylene glycol (PPG) blocks of different sizes and molecular weights (PEG-PPG-PEG, Pluronics) have gained much interest as carriers for PSs [8,23,24,25,26,27]. Recently, we have shown that Ce6-derived PSs, including Ce4, are well-encapsulated into PVP and into biodegradable BCMs formed by Kolliphor P188 (KP, Figure 1C) [28,29,30,31]. Both carriers stabilize the chlorin in an aqueous solution by preventing aggregation and maintaining its photophysical properties, and the uptake of Ce4 into HeLa cells could be proved by fluorescence microscopy [31].

Both PS (Ce4) and NP (PVP, KP) are biocompatible and have very low dark toxicity [17,20,31,32], which is required to avoid side effects while at the same time ensuring a controlled cytotoxic effect of the PS that is directed by light irradiation for localized therapy. Typically, the dark toxicity of PSs is assessed under the exclusion of light by common cytotoxicity assays like the Alamar Blue [33,34] or MTT test (using a *m*ethyl-*t*hiazol-*t*etrazolium dye) [34,35] that are based on the activity of cellular enzymes. However, the perturbation of the physiologic state of the cells, i.e., their metabolic response toward treatment with PSs in the dark, is rarely studied. Similarly, the role of the nano-sized polymeric PS-delivery system must be evaluated and better understood: How does the carrier affect the biocompatibility, overall cellular homeostasis, and molecular processes triggered by the PS? How does the nature of the carrier modulate these processes? In vitro model studies using cultured human cells are suitable and easily accessible to address the intracellular fate of PS in the dark and its polymeric carrier [36,37]. Interactions of drug delivery systems with cells encompass their cell uptake pathways, intracellular distribution, and induction of specific cell metabolite alterations. Understanding these key processes allows us to assess their in vivo tolerability, toxicity, and efficacy in transporting their payloads to the target [36].

High-resolution magic angle spinning (HR-MAS) NMR spectroscopy can be used to study intracellular small molecules giving rise to ^1^H NMR signals with atomic resolution. Moreover, the metabolic response of cultured living cells to interventions like drug treatment or specific growth conditions can be addressed [38,39,40]. Whole-cell spectra are evaluated for qualitative alterations and quantitative, altered metabolite ratios based on multivariate analysis. This metabolomic approach is an emerging technique applied in nanotoxicological studies to identify alterations in endogenous small metabolites induced by NPs [41]. It has already been proven as an efficient tool for nanotoxicological investigations employing NMR analysis of cell extracts [42,43] and culture media to determine the extracellular metabolites [44] or HR-MAS NMR of whole cells [45].

PVP and KP micelles represent two delivery systems with different chemical compositions and structures, which in turn modify the dynamic properties of the corresponding systems, including Ce4 release and reactivity toward proteins [31]. In this study, we aim to address the question of how PVP and KP-micelles affect the intracellular fate of Ce4 and the metabolic response of cancer cells following treatment with Ce4 alone or encapsulated into the carriers. For this, HR-MAS NMR spectroscopy was applied to cultured HeLa cells following incubation with the different test media in the dark. The results allow us to assess the extent of physiological interference of the PVP and BCM-based carriers as well as the porphyrinic PSs, that are non-toxic in the dark, on a molecular or even atomic level. Knowledge of the metabolic perturbation will help predict the in vivo tolerability of Ce4 or related PSs, including the impact of PVP or BCM carriers in the dark during the accumulation period preceding PDT.

## 2. Materials and Methods

### 2.1. Chemicals

Ce4 was purchased from Frontier Scientific (Logan, UT, USA). PVP (average MW = 10 kDa) and Kolliphor P188 (KP, average MW = 8.4 kDa) were obtained from Sigma-Aldrich (Buchs, Switzerland). The deuterated solvents DMSO-d6 (99.95%) and D_2_O (99.9%) were purchased from Cambridge Isotopes Laboratories, Inc. (Andover, MA, USA). Phosphate-buffered saline (PBS, 50 mM, pH = 7.3) was prepared by mixing aliquots of 50 mM solutions of KH_2_PO_4_ and Na_2_HPO_4_ (provided by Sigma-Aldrich, Buchs, Switzerland) in H_2_O or D_2_O containing 0.9% NaCl.

### 2.2. Cell Culture

The human HeLa cervical cancer cell line was kindly provided by the group of Prof. Mühlemann, University of Bern. For cell culture, phenol red-free Dulbecco’s Modified Eagle Medium (DMEM) was prepared by diluting DMEM powder (D5030-1L, Sigma) in 1 L distilled water and adding L-glutamine, sodium bicarbonate, D-glucose, and sodium pyruvate (all Sigma-Aldrich, Buchs, Switzerland). The growth medium was supplemented with 10% fetal calf serum (FCS), 100 μg/mL streptomycin (S), and 100 IU penicillin (P, DMEM+/+). The cells were grown at 37 °C in 5% CO_2_ under a humid atmosphere. Trypsin-EDTA (T/E) with 0.5 g/L trypsin (1:250) and 0.2 g/L EDTA·4Na, FCS, P, and S was obtained from Amimed (BioConcept, Allschwil, Switzerland). The culture medium was sterile-filtered through 0.1 µm polyethersulfone (PES) membranes (Nalgene™ Rapid-Flow™, Thermo Fisher Scientific, Fisher Scientific AG, Reinach, Switzerland). 

### 2.3. Cellular Uptake by Flow Cytometry

Relative quantitative uptake of Ce4 alone and combined with PVP and KP into HeLa cells was measured by applying flow cytometry.

Time-dependent uptake curves were recorded using the Amnis Image Stream^®X^ Mark II imaging flow cytometer (Luminex Corporate, Austin, TX, USA). HeLa cells were incubated with loading media containing either no additives (controls) or 5 μM Ce4 with and without PVP (16.7 μM) or KP (3 mM) for 0.5 h, 1 h, 2 h, and 3 h. The incubation was stopped by removing the loading media, followed by washing, trypsinization, and resuspension of the cells in DMEM+/+. After the addition of PSs, all sample preparation steps were performed under the exclusion of light. Laser excitation was performed at λ = 405 nm (2 mW) and fluorescence detection at λ = 642–745 nm in addition to bright-field and side-scattering detection. Data were processed using IDEAS^®^ 6.2 software (Amnis Corporation, Seattle, WA, USA). On a subpopulation of single spherical cells, the normalized (by number of pixels) mean fluorescence intensity per cell was determined, and for all sample classes, the mean fluorescence intensity of all events (8000–10,000 cells) for each class and incubation time was plotted as a function of time.

Dose-dependent uptake was recorded using a SORP LSR II flow cytometer (BD Biosciences, Franklin Lakes, NJ, USA) and FACSDiva software, Version 8.0.1 (BD Biosciences). HeLa cells were incubated with loading media containing either no additives (controls) or Ce4 at concentrations of 10 μM, 25 μM, and 62.5 μM each with and without PVP (208.3 μM) or KP (3 mM) for 2 h. All samples were prepared in triplicate. After incubation, the cells were excited as described above for the time-dependent uptake curves. A laser with a wavelength of 407 nm was used for excitation, and the Brilliant Violet filter (660/20 nm, BV650) was used to measure fluorescence emission. Ten thousand events were recorded and replicated for each sample condition. Data analysis was performed using Flowing Software (version 2.5.1, Turku Bioscience, Turku, Finland). Side- and forward-scattering areas were measured to identify intact single cells.

### 2.4. Sample Classes for ^1^H HR-MAS NMR Spectroscopy

For the ^1^H HR-MAS NMR spectroscopic study, HeLa cells were incubated with “loading media”, i.e., FCS- and P/S-free culture media (DMEM−/−) containing (i) Ce4 (10 μM), (ii) Ce4 (25 μM), (iii) Ce4 (62.5 μM), (iv) Ce4-PVP (10 μM, 208.3 μM), (v) Ce4-PVP (25 μM, 208.3 μM), (vi) Ce4-PVP (62.5 μM, 208.3 μM), (vii) only PVP (208.3 μM), (viii) Ce4-KP (10 μM, 3 mM), (ix) Ce4-KP (25 μM, 3 mM), (x) Ce4-KP (62.5 μM, 3 mM), (xi) only KP (3 mM), and (xii) and (xiii) pure PBS as controls (two times). Each of the samples (i–xiii) was individually prepared from one culture flask in triplicate, resulting in 39 (3 × 13) cell samples in total. The sample groups and their labels are summarized in Table 1.

### 2.5. Preparation of the Loading Media i–xiii

For the loading media, the following stock solutions were prepared: 2 mM Ce4 in PBS (H_2_O) containing 10% DMSO, 60 mM KP in PBS (H_2_O), and 20.83 mM PVP in PBS (H_2_O). Aliquots of these stock solutions were mixed with DMEM−/− culture medium to yield final concentrations of Ce4 (10 µM, 25 µM, 62.5 µM), KP (3 mM), and PVP (208.3 µM). Since Ce4 required 10% DMSO for dissolution to prepare the stock solution, the same amount of DMSO was also added to all other samples. Each loading medium contained the same final amount of 10.9% PBS and 0.3% DMSO, below the toxic DMSO concentration for HeLa cell cultures [46,47]. The final concentration of KP (3 mM) had to be chosen such that it was above the critical micelle concentration (cmc) of KP to ensure the existence of KP-micelles (0.45 mM in PBS [29]). In the case of PVP, the concentration of 208.3 µM was selected to ensure sufficient loading capacity for the highest Ce4 concentration of 62.5 µM corresponding to a molar ratio of 3:10 (Ce4:PVP) [31]. The loading media were sterile-filtered through 0.1 µm PES membranes (Nalgene™ Rapid-Flow™, Thermo Fisher Scientific) before incubation.

### 2.6. Preparation of Cell Samples for ^1^H HR-MAS NMR Spectroscopy

Initially, 3 batches of HeLa cells (labelled “a”, “b”, and “c”) were grown in six 75 cm^2^ culture flasks in 10 mL DMEM+/+ to approximately 80% confluence to provide enough cell material for 13 samples (i–xiii) per batch. For each sample, HeLa cells were seeded at ~0.75 × 10^6^ cells/mL in 25 cm^2^ flasks using 5 mL (~3.75 × 10^6^ cells per sample) DMEM+/+ and allowed to grow for 24 h. Subsequently, the culture medium was removed, and the cells were washed with PBS (H_2_O). Beginning with the addition of the loading media, the cell culture samples and all work-up processes were strictly excluded from light exposure to avoid phototoxic reactions of the PS. For this, the culture flasks and Falcon tubes were wrapped with alumina foil and the light was turned off in the laboratory during cell handling. After adding 5 mL of loading medium, the cells were incubated for 2 h at 37 °C in a 5% CO_2_ atmosphere. Then, the loading medium was removed, the cells were washed with PBS (H_2_O), and they were harvested by trypsinization. The cells were transferred to 15 mL Falcon tubes and centrifuged at 2000 rcf for 5 min. The supernatant was removed, and the cell pellet was washed three times with PBS (H_2_O), finally resuspended in 20 µL D_2_O-based PBS, and transferred to a black Eppendorf vial. To stop the metabolism, cells were lysed by three alternating cycles of ultrasonication (45 s) and submersion into liquid nitrogen (15 s) [48]. Frozen samples were stored at −80 °C until measurement.

On the day of use, the cell sample was thawed within 2 min and heated for 20 min at 70 °C to inactivate enzymes and ensure stability during the measurement time [48]. Finally, the lysed and heated cell suspension (~5 × 10^6^ cells in PBS-D_2_O, ~50 mg) was transferred to a 50 μL zirconium oxide MAS rotor and the rotor was briefly centrifuged (~5 s) to avoid any air bubbles before closing it with a PTFE Teflon insert and KelF rotor cap. The amount of sample inside the rotor was controlled by weight.

### 2.7. ^1^H HR-MAS NMR Spectroscopy–Data Acquisition

All HR-MAS NMR experiments were performed on a Bruker Avance II spectrometer (Bruker BioSpin, Fällanden, Switzerland) operating at a resonance frequency of 500.13 MHz for ^1^H nuclei. The instrument was equipped with a 4 mm HR-MAS dual inverse ^1^H/^13^C probe (Bruker BioSpin) with a magic angle gradient. The samples were inclined around the magic angle (54.7°), spun at 5000 Hz, and the temperature was set to 273 K (nominal). Data acquisition was performed with the Bruker software TopSpin (version 3.2, patch level 5). All samples were measured in random order, and the time between thawing and the start of the acquisition was kept constant (47 min ± 8 min). For each sample, the following spectra were recorded: (i) T_2_-edited ^1^H spectra using the 1D PROJECT (Periodic Refocusing Of J Evolution by Coherence Transfer [49]) pulse sequence with water presaturation (“*projectpr1d*”) and a T_2_ filter of 400 ms to suppress broad components with short T_2_ relaxation times. In total, 512 transients were accumulated for each 1D ^1^H spectrum, applying a spectral width of 6009.6 Hz, a data size of 32 k points, an acquisition time of 2.73 s, and a relaxation delay of 4 s. (ii) ^1^H spectra using a 1D NOESY (Nuclear Overhauser Enhancement SpectroscopY) pulse sequence (“*noesygppr1d*” from the Bruker pulse-program library) with spoil gradients for water suppression. In total, 256 transients were acquired over a spectral width of 5000 Hz with a data size of 32 k points, a mixing time of 10 ms, an acquisition time of 3.28 s, and a relaxation delay of 4 s. (iii) 1D ^1^H diffusion edited spectra applying the 1D DOSY (Diffusion Ordered SpectroscopY) stimulated echo pulse sequence (“*ledbpgppr2s1d*” from the Bruker pulse-program library) with longitudinal eddy current delay of 5 ms, bipolar gradients for diffusion, 2 spoil gradients, and presaturation of the water resonance. Each spectrum was recorded with 256 transients, a spectral width of 6009.6 Hz, a data size of 32 k points, and a relaxation delay of 2 s. To suppress small fast diffusing metabolites, a gradient was applied at 40% of the maximal gradient strength corresponding to 13.5 G/cm, applying a sine-shaped gradient. The diffusion time Δ was set to 50 ms and the gradient length δ to 6 ms.

On selected samples, 2D DOSY spectra (“*ledbpgppr2s*” from the Bruker pulse-program library) were acquired using the same parameters as for the 1D DOSY spectra applying an incremented linear gradient ramp from 5 to 95% over 64 steps with 16 scans per increment. 

To support resonance assignments, 2D ^1^H^1^H TOCSY (TOtal Correlation SpectroscopY) spectra were acquired on a representative subset of samples applying the DIPSI2 pulse sequence (“*dipsi2phpr*” from the Bruker pulse program library) with presaturation of the water resonance. For the TOCSY experiment, 128 transients were acquired with a data size of 2048 × 256 (F2, F1) over a spectral width of 5000 Hz in both dimensions (F2, F1). The relaxation delay was set to 1.2 s, and the TOCSY mixing time to 74.8 ms. 

### 2.8. Processing of ^1^H HR-MAS NMR Spectra

Spectral processing was performed using Bruker Topspin software (version 3.5b, patch level 7). For the 1D PROJECT and 1D NOESY spectra, exponential multiplication of the FIDs with a line-broadening factor of 1.0 Hz was performed, followed by phasing and baseline correction. The baseline offset was corrected (zero-order polynomial) for the whole spectral range, and for the 1D PROJECT spectra, a 5th-order polynomial baseline correction was also applied to the spectral region between 5.1 ppm and 10 ppm. Chemical shifts were referenced to the -CH_3_ resonance of creatine (Cre) at δ = 3.03 ppm. For the 1D DOSY spectra, an exponential multiplication of the FIDs with a line broadening factor of 5 Hz was applied. The spectra were phased, baseline corrected (zero-order polynomial over the whole spectral range), and referenced to the -CH_3_ resonance of choline (Cho) at δ = 3.208 ppm. 2D DOSY spectra were processed using the Bruker software “Dynamics Center” (version 2.6.2) by fitting the peak intensities of selected peaks as a function of gradient strength with a bi-exponential fitting function. The TOCSY data were processed by zero filling (to 512) in F1 and using shifted squared sine window functions in both dimensions prior to 2D Fourier transformation.

Assignment of the resonances was based on ^1^H^1^H TOCSY spectra (Figure S1A–C), spectral databases (Human Metabolome Database, HMDB [50] and in-house database), and literature data [45,51,52,53,54].

### 2.9. Data Analysis

Multivariate analyses of the 1D PROJECT spectra were performed using MATLAB R2019b (The MathWorks, Inc., Natick, MA, USA), PLS Toolbox (version 8.8.1, Eigenvector Research, Inc., Manson, WA, USA), and Excel (Microsoft Office 365 ProPlus, Redmond, WA, USA).

The 39 ^1^H HR-MAS NMR spectra were subdivided in the spectral range between 0.8 and 9.5 ppm into 259 individually sized buckets (integral regions), excluding regions with noise, with the residual water resonance (4.8–5.25 ppm), ethanol resonance, and PEG-CH_2_ and PPG-CH_3_ resonances of KP (1.11–1.2 ppm and 3.62–3.74 ppm). Bucket selection was performed on an overlay of all spectra, followed by integration in the corresponding regions using TopSpin software. The integrals were imported into PLS-Toolbox for further analysis of the 39 × 259 data matrix. To account for differences in sample amounts, probabilistic quotient normalization (PQN) [55] was applied to the integrals of each sample. Furthermore, preprocessing included mean centering and Pareto scaling. The data were subdivided into 12 classes according to (i)–(xii) (see Section 2.4), with the six control samples (xii, xiii) belonging to one class. Unsupervised principal component analysis (PCA) was performed to probe for sample clustering. Partial least squares discriminant analysis (PLS-DA) was used to calculate a model for distinguishing the spectra based on the assignment to 12 classes. Orthogonal partial least squares analysis (oPLS) was applied to calculate a regression model for predicting concentration-dependent metabolite alterations. Cross-validation for PCA, PLS-DA, and oPLS was performed by Venetian blinds with 10 splits and a blind size of 1. Model statistics (root mean square error of calibration/cross-validation, RMSEC/RMSECV) and classification errors were calculated for PCA and PLS-DA. Validation metrics R^2^ and Q^2^ were calculated for the linear oPLS regression model. Univariate analysis was performed on single metabolites (representative buckets/integrals) in Excel.

## 3. Results and Discussion

### 3.1. Cell Uptake of Ce4 and the Polymeric Carriers PVP and KP

**Figure 2:**
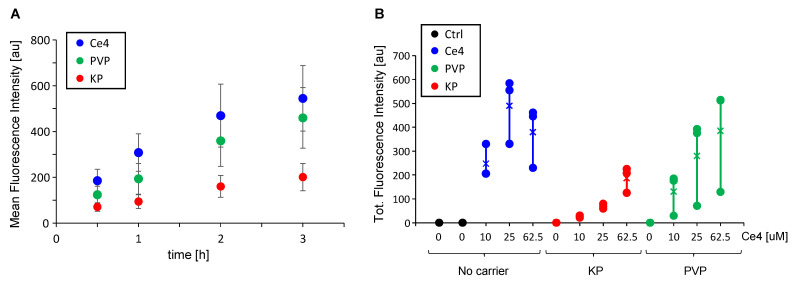
Chlorin e4 (Ce4) uptake into HeLa cells measured as intrinsic fluorescence following addition of Ce4 alone (blue), encapsulated into polyvinylpyrrolidone (PVP, green), or Kolliphor P188 (KP, red): (**A**) as function of incubation time (5 μM Ce4, 16.7 μM PVP, 3 mM KP) and (**B**) as function of Ce4 concentration (208.3 μM PVP, 3 mM KP, 2h incubation).

To quantify the cellular uptake of Ce4 depending on its formulation, i.e., either in its free form or encapsulated into PVP or KP micelles, HeLa cells were measured by flow cytometry following treatment with the corresponding samples added to the culture medium. For this, the intrinsic fluorescence intensity of Ce4 associated with the HeLa cells was recorded. Both time-dependent uptake for a fixed Ce4 concentration of 5 μM (Figure 2A) and concentration-dependent uptake during a fixed time of 2 h (same Ce4 concentrations as applied in the HR-MAS NMR study, Figure 2B) were measured. Ce4 uptake by HeLa cells was time-dependent and marginally leveled off after approximately 2–3 h. It was slightly reduced when Ce4 was encapsulated in PVP and distinctly reduced in the presence of KP micelles (Figure 2A). This reduction was also found for the Ce4 concentration range of 10–62.5 μM when applied with carriers (Figure 2B). In the presence of PVP or KP, cell uptake increased with Ce4 concentration without reaching saturation and was always lower than Ce4 in its free form, except for Ce4-PVP at 62.5 μM Ce4.

In the absence of carriers, a fluorescence drop was observed at the highest Ce4 level (62.5 μM). At this concentration, chlorin aggregation becomes prevalent, leading to pronounced fluorescence quenching [31] so that the measured fluorescence intensity no longer correlates with the actual intracellular Ce4 concentration. In contrast, these aggregation and quenching effects are prevented through the encapsulation of Ce4 into PVP or KP, where it exists in the monomeric state [31].

Reduced cellular drug uptake from Pluronic micelles has been previously reported [31,56] and discussed in detail with respect to a single incubation time [31]. Possible causes for a reduced uptake of PEGylated lipid NPs by tumor cells have been related to steric hindrance and blocking of ligands at the cell surface through PEG chains. These drawbacks accompanying the advantages of PEGylated lipid NPs were denoted as the “PEG-dilemma” [57,58]. Under in vivo conditions, the disadvantage of reduced cell uptake is partly compensated by the enhanced stability and prolonged lifetime of the drug-loaded NPs during the transport in the bloodstream. This is mainly due to the stealth effect of the PEG corona preventing the opsonization process [59] and the EPR-mediated accumulation of PEGylated NPs in tumor tissue [11]. However, these effects are not reflected in the in vitro cell culture model. 

### 3.2. The Metabolic Profile of Treated and Untreated HeLa Cells: T_2_-Edited ^1^H HR-MAS NMR Spectra

**Figure 3:**
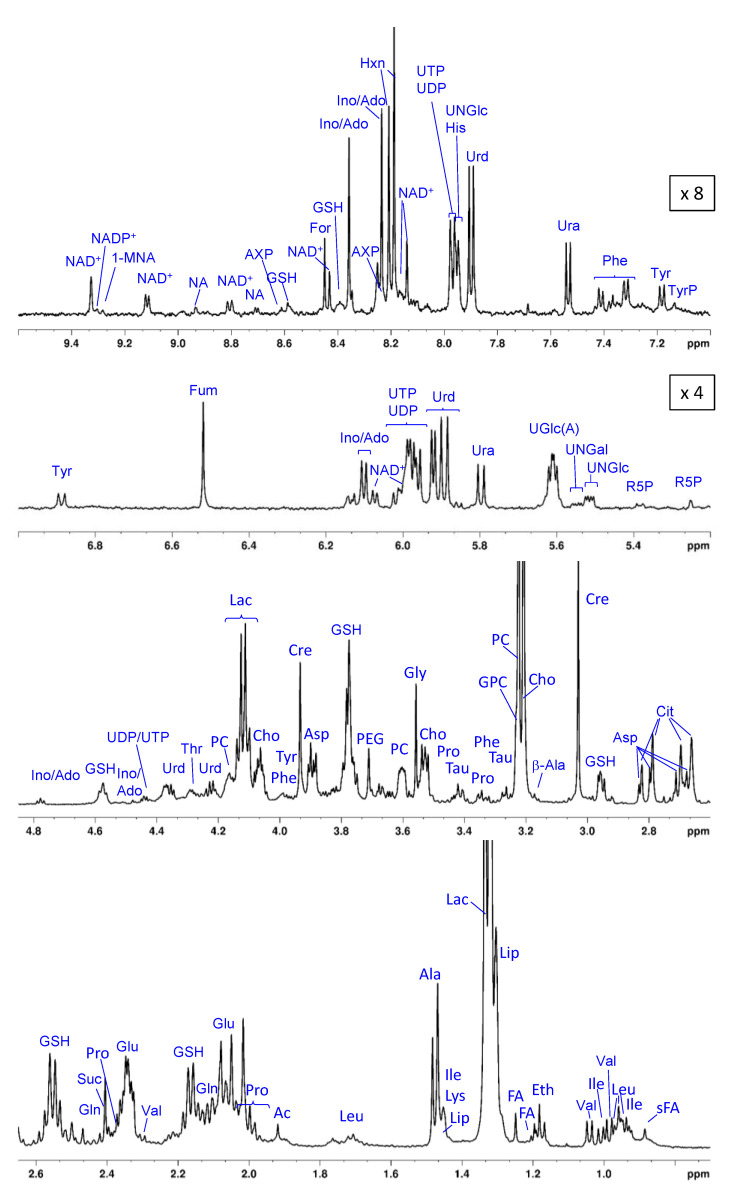
^1^H high-resolution magic angle spinning NMR summed spectrum (T_2_-filtered) of lysed HeLa cell suspension in phosphate-buffered saline with resonance assignments. Spectral regions of 5.2–7.0 ppm and 7.0–9.6 ppm were scaled up by a factor of 4 and 8, respectively. Ac: acetate; Ala: alanine; β-Ala: β-alanine; Asp: aspartate; AXP: adenosine phosphate; Cho: choline; Cit: citrate; Cre: creatine; Eth: ethanol; FA: fatty acids; For: formic acid; Fum: fumarate; Gln: glutamine; Glu: glutamate; Gly: glycine; GPC: glycerophosphocholine; GSH: glutathione; His: histidine; Hxn: hypoxanthine; Ile: isoleucine; Ino/Ado: inosine/adenosine; Lac: lactate; Leu: leucine; Lip: lipids; Lys: lysine; 1-MNA: 1-Methylnicotinamide; NA: nicotinamide; PC: phosphocholine; PEG: polyethylene glycol; Phe: phenylalanine; Pro: proline; R5P: ribose-5-phosphate; sFA: fatty acids (saturated); Suc: succinate; Tau: taurine; Thr: threonine; Tyr: tyrosine; TyrP: tyrosine-containing peptide; UDP: uridine diphosphate; UGlc(A): UDP-glucose/UDP-glucuronic acid; UNGal: UDP-N-acetyl-galactosamine; UNGlc: UDP-N-acetyl-glucosamine; Ura: uracil; Urd: uridine; UTP: uridine triphosphate; Val: valine.

In Figure 3, a summed T_2_-filtered (PROJECT) ^1^H HR-MAS NMR spectrum of all lysed HeLa cell suspensions in PBS is shown. Typically, with the relaxation filter, underlying broad peaks deriving from large molecules like proteins, lipids, or polysaccharides are suppressed, facilitating the analysis of small molecules that give rise to sharp resonances. Each single spectrum provides a metabolic snapshot of the cells following their different exposures to the various test media (i–xiii). A total of 53 compounds could be identified, which are summarized in Table 2, along with their abbreviations, chemical shifts, and multiplicities. The most prominent peaks originate from cell metabolites such as Lac, mobile lipids (Lip), the choline-containing compounds Cho, PC, and GPC, Cre, and several amino acids like Gln, Leu, Ile, and Val. The spectral region between 5 ppm and 10 ppm that typically exhibits resonances of much lower intensity is dominated by contributions from nucleosides, nucleotides, and related components such as UDP- and UTP derivatives. Among those, the nucleotide sugars UNGlc, UNGal, and UGlcA (most likely overlapping with UGlc) are assigned based on the resonances deriving from their anomeric protons around 5.6 ppm with the characteristic multiplet structure [52]. The assignment of the purine-derived metabolites Ino and Ado remains ambiguous since the chemical shift values of the two single purine resonances are hard to distinguish (annotation Ino/Ado). In addition, there is a pronounced pH dependence in the chemical shifts of adenine-derived nucleotides (AXP), as previously discussed [53]. In the current study, AXP, most likely AMP [53,60], and UMP were only observed in the spectra of samples treated with Ce4. Furthermore, among the compounds listed in Table 2, some were only detectable in the presence of KP or without applying a T_2_ filter (see Section 3.3 below).

### 3.3. The Metabolic Profile of Treated and Untreated HeLa Cells: Diffusion-Edited ^1^H HR-MAS NMR Spectra

**Figure 4:**
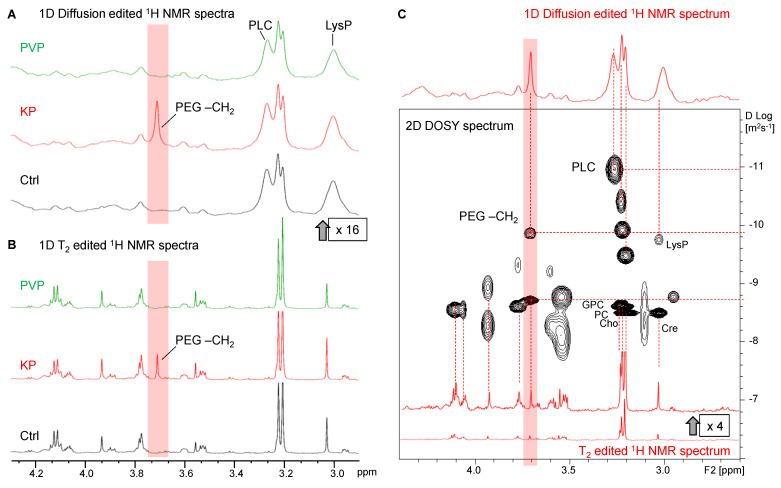
(**A**) HR-MAS ^1^H 1D diffusion-edited NMR spectra (scaled up by a factor of 16) and (**B**) HR-MAS ^1^H-T_2_-edited (PROJECT) NMR spectra of lysed HeLa cell suspensions in phosphate-buffered saline (PBS). Cells incubated with PBS as controls (Ctrl, black), Kolliphor P188 (KP, red), and polyvinylpyrrolidone (PVP, green). The polyethylene glycol (PEG)-CH_2_ resonance was only visible in cells incubated with KP and is highlighted in red. (**C**) HR-MAS ^1^H 2D DOSY spectrum of a cell suspension incubated with KP; 1D diffusion- and 1D T_2_-edited projections are shown in red. The PEG-CH_2_ resonance corresponds to a slow and a fast diffusing component (highlighted in red). Cho: choline; Cre: creatine; GPC: glycerophosphocholine; LysP: lysine-containing peptide; PC: phosphocholine; PEG: polyethylene glycol; PLC: phosphatidylcholine.

In 1D ^1^H DOSY, small metabolites with a larger diffusion coefficient can be suppressed by adjusting the diffusion time and gradient strength during acquisition so that only the broad resonances of slowly diffusing macromolecular components remain visible. The effects of T_2_ relaxation and diffusion filters on the ^1^H NMR spectra are visualized in Figure 4 and Figure S2 and contrasted to unfiltered 1D NOESY spectra covering both sharp and broad components (Figure S2).

#### 3.3.1. Lipids and Oligopeptides

Primarily lipid resonances remained visible in the 1D DOSY spectra used in addition to TOCSY spectra (Figure S1) to assign the corresponding lipid moieties (Figure S2 and Table 2). This also included the characteristic broad -N^+^(CH_3_)_3_ PLC resonance of phospholipids at 3.27 ppm [61,62]. In addition, oligopeptides give rise to broader resonances mainly visible in diffusion-edited spectra, notably Tyr- and Lys-containing peptides [63] (TyrP, Figure S1C and LysP, Figure 4A). TyrP and LysP have similar chemical shift values compared to their amino acid counterparts [62] and exhibit analogous cross peaks in TOCSY (Figure S1).

#### 3.3.2. Visibility of Intracellular KP

Notably, in all spectra of HeLa cell samples treated with KP (alone or with encapsulated Ce4), the presence of the PEG-CH_2_ resonance was clearly visible at 3.7 ppm (Figure 4A,B). This indicates cell internalization of the block copolymer-based carrier KP. The fact that the PEG resonance visibility is maintained in the diffusion-edited spectra points toward the presence of micelles or polymer aggregates inside the cell that may be coexistent with monomers. Since BCMs disintegrate upon dilution below their cmc, the KP concentration in the incubation media was selected well above the cmc to maintain micelles in the current study. To further investigate the diffusion properties associated with the PEG-resonance at 3.7 ppm, 2D DOSY spectra were recorded, and the gradient strength-dependent peak intensity decay curves of selected resonances were analyzed applying bi-exponential fitting (Figure S3). A corresponding 2D DOSY spectrum is shown in Figure 4C for the spectral region between 2.5 ppm and 4.5 ppm, with a 1D diffusion-edited spectrum as projection (on top) showing the slow diffusing components and a T_2_-edited spectrum as projection (on the bottom) showing mainly the fast-diffusing components. In the DOSY spectrum, the diffusion coefficient (D-value) is provided along the y-axis in the logarithmic scale. The decay curve of PEG-CH_2_ was clearly bi-exponential, indicating the existence of both smaller and larger molecular assemblies, potentially whole micelles. Similarly, the resonances assigned to choline-containing compounds represent small molecules (Cho, PC, and GPC) that dynamically behave like Cre as well as larger components, among which the PLC resonance of phospholipids gives rise to the smallest D-value.

Uptake mechanisms of polymeric micelles are not yet completely understood, and often only their payload, i.e., the drug molecules, are traced inside the cell and not the carrier material itself. The cell uptake pathway of a related PEG-PPG-PEG polymer, namely Pluronic P85, was reported to be strictly concentration-dependent [64]. While the single building blocks were internalized via caveolae-mediated and caveolae- and clathrin-independent endocytosis, P85 micelles were exclusively taken up via the classical clathrin-mediated endocytosis [13,64]. Moreover, the possibility of disintegration of polymeric micelles upon cell internalization was also discussed, leading to the separation of the polymer and drug molecules before uptake [65].

#### 3.3.3. Invisibility of Intracellular PVP

Unlike KP, no PVP resonances were visible in the spectra of PVP-treated cells. This may be due to the fact that the applied concentration of PVP was more than 10-fold lower than that of KP. In addition, the large and slowly tumbling PVP molecular network leads to fast T_2_-relaxation associated with broader line widths. Consequently, the broad PVP resonances are suppressed in the T_2_-edited spectra, as was demonstrated on a pure PVP sample in PBS with and without the T_2_ filter used in this study (Figure S4). However, when applying the diffusion filter, the PVP resonances should be traceable if they exist in solution at sufficient concentrations (Figure S4). Therefore, to probe for any intracellular NMR visibility of PVP, additional samples of HeLa cells were incubated with a high concentration (30 mM) of PVP and compared to cells incubated with equally concentrated KP in the culture medium. However, even at this high concentration, PVP resonances were not detectable in the diffusion-edited cell spectra (Figure S5). On the other hand, efficient uptake of PVP encapsulated Ce4 into HeLa cells was proven by flow cytometry in this study (Figure 2) and previously by confocal laser scanning microscopy [31]. While PVP may not be internalized, only releasing its payload into the HeLa cells, this assumption is rather unlikely according to literature reports on the cellular uptake of NPs. As an example, it could be shown that PVP covalently bound to hypericin was internalized into HeLa cells [66], and for octadecyl-modified PVP NPs, dynamin-dependent endocytosis was suggested as a major uptake mechanism besides passive diffusion through the cell membrane [65,67,68]. Accordingly, it is most likely in the present work that intracellular PVP is associated with cellular macromolecular structures like proteins or membranes, further increasing the line-broadening of the PVP resonances, leading to their NMR-invisibility.

#### 3.3.4. Indirect Detection of Intracellular Ce4

The direct observation of NMR resonances from intracellular Ce4 is concealed due to low concentration and possibly to Ce4 association with large molecules leading to pronounced line broadening. For example, previously, it was shown that Ce4 binding to human serum albumin (HSA) is accompanied by the loss of visible Ce4 proton resonances in buffer solution [31]. A similar effect was observed upon insertion of Ce6 derivatives into phospholipid bilayers of DOPC (di-oleoyl-phosphatidylcholine) vesicles [61]. However, the strong ring current of porphyrinic macrocycles in magnetic fields [69] can induce considerable changes in NMR chemical shifts of biomolecules in close proximity, allowing indirect porphyrin detection [70]. 

**Figure 5:**
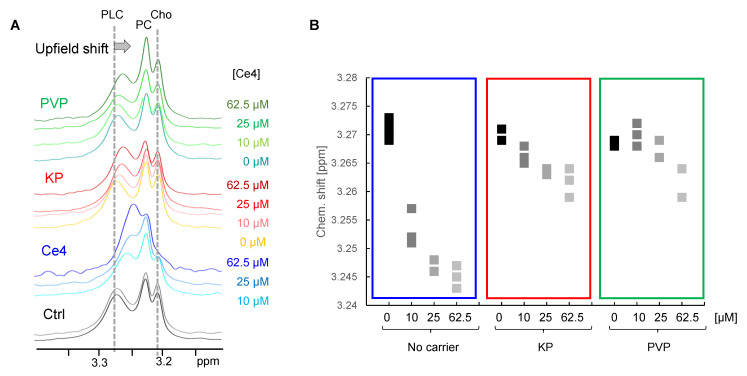
(**A**) HR-MAS ^1^H diffusion-edited NMR spectra (1D-DOSY) of lysed HeLa cell suspensions in PBS incubated with different concentrations of chlorin e4 (Ce4) without carrier or encapsulated into Kolliphor P188 (KP) or polyvinylpyrrolidone (PVP). Shown is the spectral region where the choline-containing resonances appear. The PLC resonance exhibits upfield shifts; (**B**) chemical shift of the PLC-resonance as a function of condition (Ce4 concentration and carrier). Cho: choline; PC: phosphocholine; PLC: phosphatidylcholine.

In the current study, such an induced chemical shift change could be observed in the diffusion-edited spectra of cells incubated with Ce4. Whereas the Cho and PC resonances remained unchanged, a clear upfield shift of the PLC N-methyl choline resonance appeared. This upfield shift correlated with Ce4 concentration and was also detectable when Ce4 was applied with KP and PVP, but to a lower extent (Figure 5A,B). This Ce4 ring current-induced shift reflects a direct interaction with cellular phospholipids, suggesting Ce4 association with phospholipid membranes. This is in agreement with the assumption that cellular membranes are the preferential intracellular localization sites of porphyrinic PSs [71,72]. Our previous model studies on externally added amphiphilic Ce6 derivatives to unilamellar phospholipid vesicles as model membranes have suggested a preferential association with the choline-containing head group regions of the phospholipids [61,73,74]. Therefore, the current approach provides the unique possibility to localize intracellular porphyrin not just on a molecular but even atomic level.

The PLC-upfield shifts observed for the different Ce4 formulations (no carrier, KP, PVP) provide additional valuable information on the intracellular impact of the carrier. The magnitude of the ring-current-induced shift depends on both the local Ce4 concentration and the spatial proximity of the chlorin macrocycle to the membrane. This seems largest for Ce4 in its free form, whereas KP and PVP have an attenuating effect, which is stronger for PVP despite higher Ce4 uptake with PVP than KP (Figure 2). Accordingly, it is more likely that the previously observed eased release of Ce4 from the micellar KP carrier [31] allows more direct association with cellular membranes than PVP. The stronger binding of Ce4 to PVP suggests that either less free Ce4 is available for direct membrane association and/or that the whole Ce4-PVP assembly binds to the membrane, leading to a spatial shielding of the chlorin ring current effect through the surrounding PVP network. Nevertheless, the presented NMR data underline the hypothesis that the nature of the carrier has a pronounced impact on the intracellular fate of the transported PS, which is expected to modulate PDT efficiency. 

### 3.4. Metabolic Analysis of Cell Spectra: Impact of Ce4, PVP, and KP

To probe the impact of Ce4 alone or encapsulated into PVP or KP micelles onto the metabolic profile of cultured HeLa cells in the dark, cells were incubated under the exclusion of light for 2 h with three different Ce4 concentrations (10 μM, 25 μM, and 62.5 μM) in pure PBS, PVP, or KP each at a constant polymer concentration in PBS. The polymers without Ce4 were also included, and cells incubated with pure PBS added to the culture media served as controls. This resulted in 12 sample classes measured in triplicates and labeled as Ce4-10, Ce4-25, Ce4-62.5, KP, KP-Ce4-10, KP-Ce4-25, KP-Ce4-62.5, PVP, PVP-Ce4-10, PVP-Ce4-25, PVP-Ce4-62.5, and Ctrl (Table 1). The spectra were subdivided into individually sized bucket regions covering single resonances as much as possible.

#### 3.4.1. PCA

**Figure 6:**
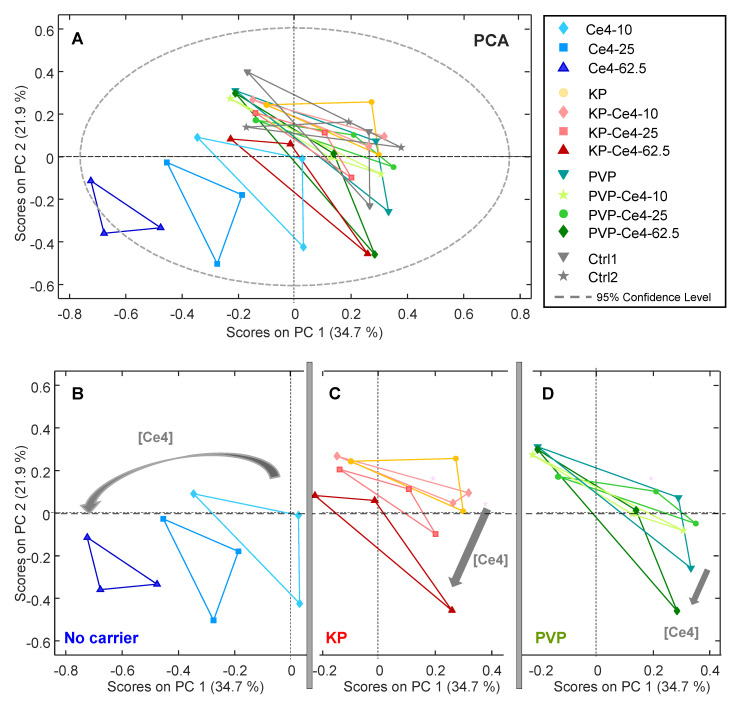
(**A**) Principal component analysis (PCA) applied to all samples and buckets from 1D PROJECT spectra (259 × 39). (**B**–**D**) Excerpts from the PCA plot (**A**) for better visibility of the subclasses: (**B**) samples without carriers, pure chlorin e4 (Ce4); (**C**) samples with Kolliphor P188 (KP) as a carrier; (**D**) samples with polyvinylpyrrolidone (PVP) as a carrier; PCA model: RMSEC: 0.014, RMSECV: 0.02, number of PCs: 5.

In the first step, unsupervised PCA was applied to all samples to make potential clustering of sample classes visible and to probe for outliers. In Figure 6A, the PCA scores plot is displayed for the first two components, PC-1 and PC-2, accounting for a total variance of 56.6%. While the samples incubated with Ce4 without carrier, shown in blue, were well-separated in the lower quarter of the plot with negative PC-1 and PC-2 scores, the remaining samples overlapped. For the carrier-free samples, there was a Ce4-concentration-dependent shift towards increasingly negative scores along PC-1. A correlation with Ce4 concentration, even though less pronounced, was also observed for the samples with KP and PVP. To make this correlation more visible, the corresponding subgroups are shown separately as excerpts of the common plot shown in Figure 6A (Figure 6B–D). These results indicate that Ce4 triggers a concentration-dependent metabolic response in HeLa cells with different characteristics provided by its form of application, i.e., free or carrier-associated. Notably, in the PCA scores plot, the data points from each of the triplicates possessed a triangular arrangement. This was caused by the associated batches for each sample within a triplicate (a, b, or c) of independently prepared HeLa cells (see Experimental Section 2.6). Accordingly, within each batch, the samples cluster and there is a difference in the metabolic profile between these clusters, mainly along PC-2 (Figure S6A). The loading plot for PC-1 and PC-2 (Figure S6B) reveals that the contributions derive from a few specific metabolites, mainly PC and Lac. This underlying difference can be explained by the variability inherent to independent biological samples causing systematic inter-batch variations sometimes encountered in metabolomic studies and referred to as “batch effect” in the literature [75]. However, despite these batch-dependent differences, a clear clustering of sample classes for each condition (Ce4 concentration and carrier material) superimposed the background variations. Therefore, this unsupervised PCA approach already reveals the presence of class-dependent metabolic alterations without the need for any background subtraction. 

#### 3.4.2. PLS-DA

**Figure 7:**
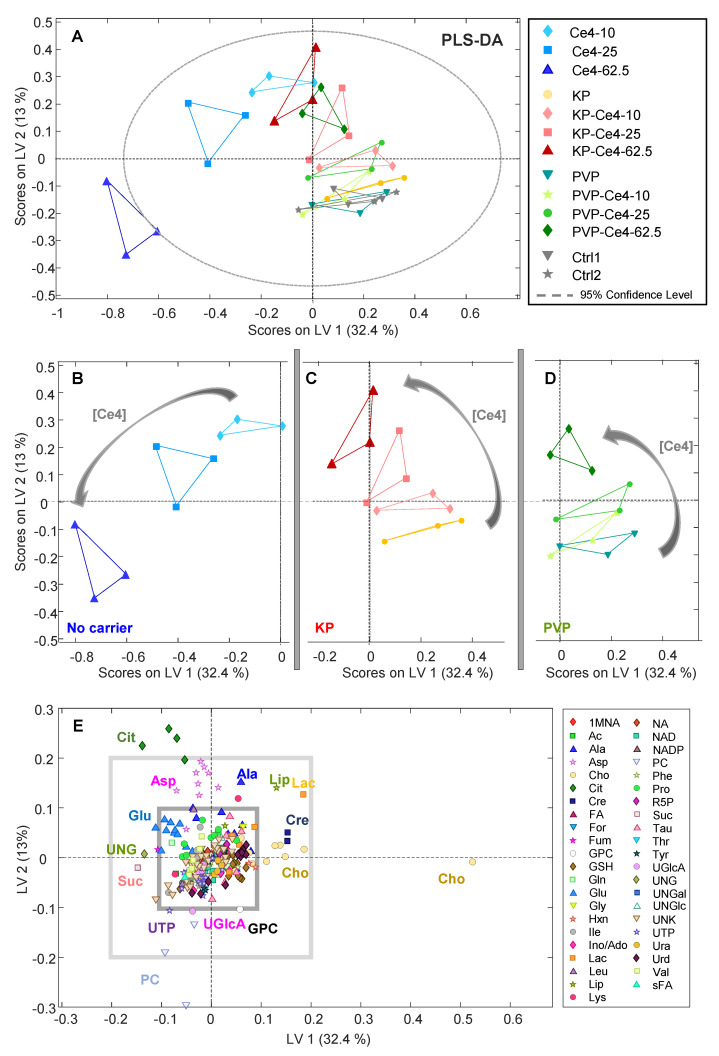
(**A**) Partial least squares discriminant analysis (PLS-DA) applied to all samples and buckets from 1D PROJECT spectra (259 × 39). Excerpts from the PLS-DA plot in (**A**) for better visibility of the subclasses: (**B**) samples without carriers, pure chlorin e4 (Ce4); (**C**) samples with Kolliphor P188 (KP) as a carrier; (**D**) samples with polyvinylpyrrolidone (PVP) as a carrier; (**E**) loading plot for LV-1 and LV-2; the squares mark the arbitrary regions of load values ±0.2 and ±0.1. Model statistics are provided in Table S1. 1-MNA: 1-Methylnicotinamide; Ac: acetate; Ala: alanine; Asp: aspartate; Cho: choline; Cit: citrate; Cre: creatine; FA: fatty acids; For: formic acid; Fum: fumarate; GPC: glycerophosphocholine; GSH: glutathione; Gln: glutamine; Glu: glutamate; Gly: glycine; Hxn: hypoxanthine; Ile: isoleucine; Ino/Ado: inosine/adenosine; Lac: lactate; Leu: leucine; Lip: lipids; Lys: lysine; NA: nicotinamide; PC: phosphocholine; Phe: phenylalanine; Pro: proline; R5P: ribose-5-phosphate; Suc: succinate; Tau: taurine; Thr: threonine; Tyr: tyrosine; UGlcA: UDP-glucose/UDP-glucuronic acid; UNG: UNGlc/UNGal; UNGal: UDP-N-acetyl-galactosamine; UNGlc: UDP-N-acetyl-glucosamine; UNK: unknown; UTP: uridine triphosphate; Ura: uracil; Urd: uridine; Val: valine; sFA: fatty acids (saturated).

In the next step, PLS-DA was performed on all samples to probe if the 12 classes could be distinguished according to the combined features of Ce4 concentration and formulation. The PLS-DA scores plot for the first two latent variables, LV-1 and LV-2, is shown in Figure 7A. The samples of the non-encapsulated Ce4 (blue) appear as the most separated group with negative scores on LV-1. The magnitude of the LV-1 score increases with Ce4 concentration. A similar but attenuated trend toward negative LV-1 scores can also be observed for KP- and PVP-encapsulated Ce4, where for each, the samples at the highest concentration lie close to the carrier-free Ce4 sample points (blue). The discrimination from the Ce4-free samples (Ctrl1, Ctrl2, PVP, and KP) that cluster in the lower right quartile of the plot is most pronounced for carrier-free (blue) followed by micellar-encapsulated (KP, red) Ce4 and is least pronounced for PVP-encapsulated Ce4 (green). In analogy to the induced PLC-resonance shift discussed in Section 3.3.4, the cell-metabolic response toward Ce4 treatment is clearly diminished by KP and even stronger by PVP encapsulation. The easier release of Ce4 from the micellar KP carrier may be responsible for the difference observed with respect to PVP. Thus, the carriers probed here, particularly PVP, have a protective role, leaving the PS inactive after cell internalization in the dark before light activation can trigger the desired cell damage. In addition, samples incubated with the carriers alone overlap with the control samples in both PCA (Figure 6) and PLS-DA (Figure 7). This indicates mild or no measurable metabolic cell response as anticipated for the pure carrier material alone.

Again, to disentangle the formulation-related subgroups, the corresponding parts of the PLS-DA plot were extracted and shown separately in Figure 7B–D for better visibility of intra-group distributions. These excerpts clarify the correlation with Ce4 concentration within each sub-group that mainly impacted LV-2 scores. Both for KP (Figure 7C) and PVP (Figure 7D), the LV-2 scores move from negative toward increasingly positive values with Ce4 concentration, whereas the trend turns in the opposite direction for carrier-free samples (Figure 7B). In Figure 7E, the two-dimensional loading plot for LV-1 and LV-2 is shown. For classifying the impact of metabolite contributions to the PLS-DA model (Figure 7A) according to their magnitude, two squares (light and dark gray) were overlaid, marking the arbitrarily selected load values of ±0.1 and ±0.2 along both dimensions (Figure 7E). According to this scale, Cho, PC, and Cit have the largest impact on separating the sample classes with load values outside the “±0.2-square”. This indicates that Cho levels are inversely correlated with the concentration of free Ce4 (main contribution along LV-1) and Cit levels are correlated with concentration of carrier-associated Ce4 or moderately increased free Ce4, respectively, whereas PC levels decrease (main contributors along LV-2). The potential meaning of these metabolic changes will be further discussed in the subsequent sections.

#### 3.4.3. oPLS Analysis of Subgroups

**Figure 8:**
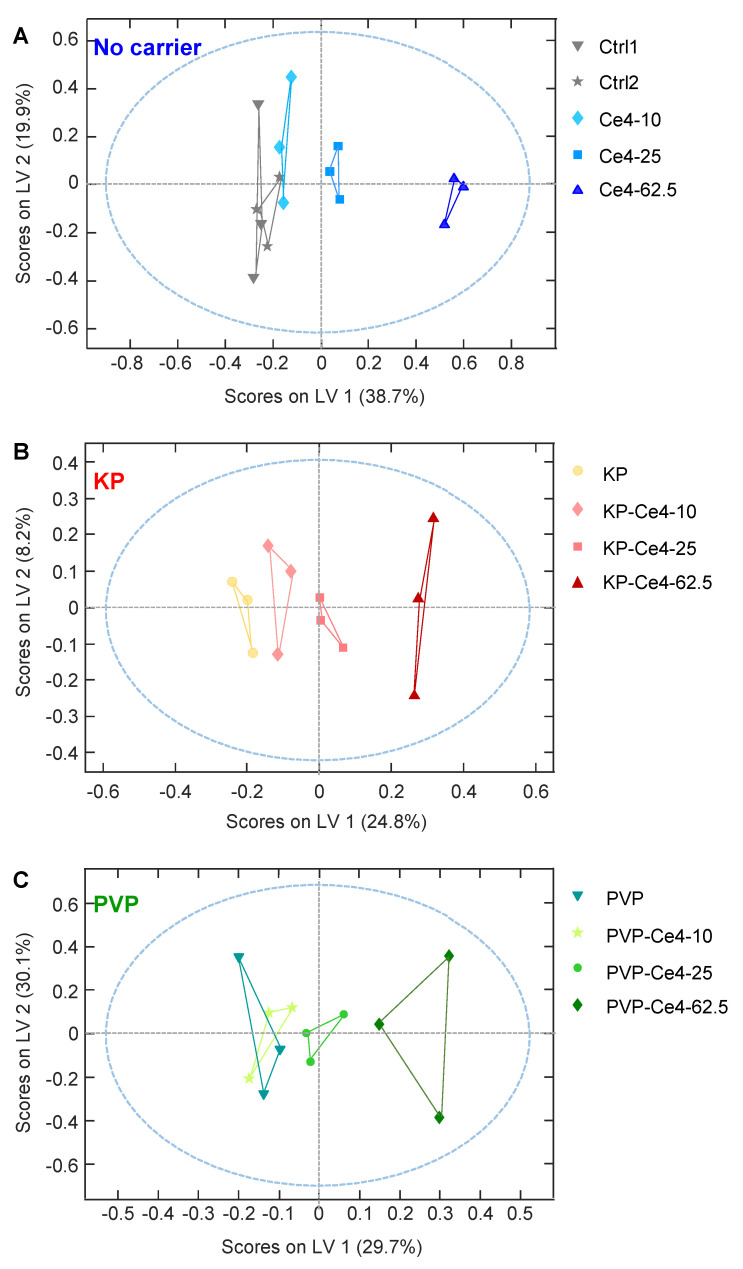
Orthogonal partial least squares (oPLS) analysis applied to samples and buckets from 1D PROJECT spectra as an X-matrix and chlorin e4 (Ce4) concentration (0, 10, 25, 62.5 μM) as a Y-matrix according to subclasses of (**A**) samples without carriers (259 × 15); (**B**) samples with Kolliphor P188 (KP) as a carrier (259 × 12); (**C**) samples with polyvinylpyrrolidone (PVP) as a carrier (259 × 12). The blue dashed line includes samples falling into the range of 95% confidence level. Model statistics: (**A**) R^2^ 0.99; Q^2^ 0.93; (**B**) R^2^ 0.97; Q^2^ 0.67; (**C**) R^2^ 0.94; Q^2^ 0.71.

**Figure 9:**
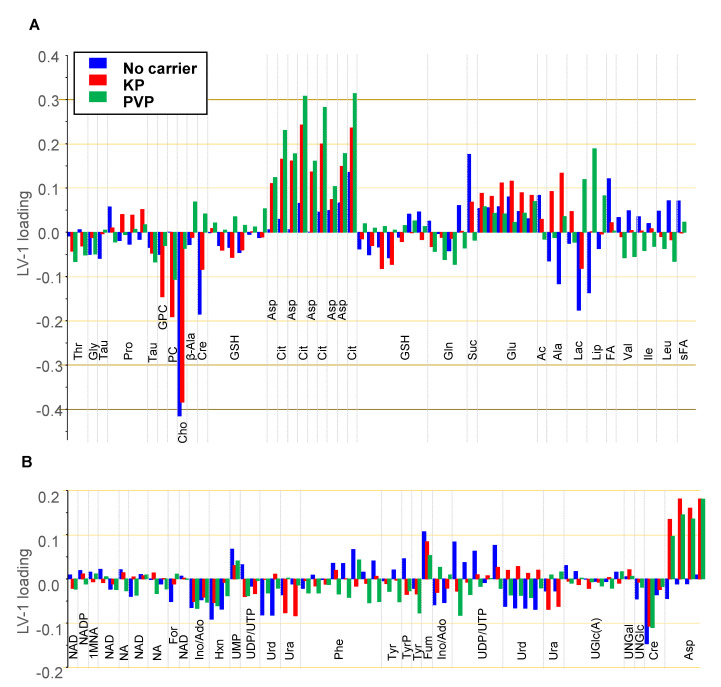
Loadings of the first oPLS components (LV-1) according to the oPLS score plots shown in Figure 8 for carrier-free samples (blue), Kolliphor P188 (KP) samples (red), and polyvinylpyrrolidone (PVP) samples (green). Loadings are shown for buckets that could be assigned to metabolites and that had little overlap. Horizontal arbitrary lines mark metabolites with respect to their contribution with load values above ±0.1, ±0.2, ±0.3, and ±0.4. (**A**) 3.6–0.8 ppm region; (**B**) 9.4–3.6 ppm region. Ac: acetate; Ala: alanine; β-Ala: β-alanine; Asp: aspartate; Cho: choline; Cit: citrate; Cre: creatine; FA: fatty acids; For: formic acid; Fum: fumarate; Gln: glutamine; Glu: glutamate; Gly: glycine; GPC: glycerophosphocholine; GSH: glutathione; Hxn: hypoxanthine; Ile: isoleucine; Ino/Ado: inosine/adenosine; Lac: lactate; Leu: leucine; Lip: lipids; 1MNA: 1-Methylnicotinamide; NA: nicotinamide; PC: phosphocholine; Phe: phenylalanine; Pro: proline; sFA: fatty acids (saturated); Suc: succinate; Tau: taurine; Thr: threonine; Tyr: tyrosine; TyrP: tyrosine-containing peptide; UDP: uridine diphosphate; UGlc(A): UDP-glucose/UDP-glucuronic acid; UNGal: UDP-N-acetyl-galactosamine; UNGlc: UDP-N-acetyl-glucosamine; Ura: uracil; Urd: uridine; UTP: uridine triphosphate; Val: valine.

To separate the impact of formulation and concentration on the metabolic cell response, oPLS analysis was performed on each subgroup (no carrier, KP, PVP, Figure 8A, Figure 8B and Figure 8C, respectively). In the subgroups comprising cell samples exposed to KP- or PVP-encapsulated Ce4, the blank samples (no Ce4) were represented by cells exposed to the pure carrier KP and PVP, respectively, thus leaving the drug dose the only variable within each group. The linear regression model was chosen to correlate the metabolic response, i.e., the magnitude and direction of metabolite level changes, with Ce4 concentration. According to the oPLS plots shown in Figure 8, there is a clear dose-dependent trend along LV-1 common to all subgroups. The scores on LV-1 shift from negative values toward increasingly larger positive values with Ce4 concentration for all three subgroups. The corresponding loadings on LV-1 are displayed as bar plots in Figure 9 for the metabolites in the spectral region 0.8–3.6 ppm (Figure 9A) and 3.6–9.4 ppm (Figure 9B). Above all, Cho levels with a load value around −0.4 have the strongest contribution with a negative correlation on the concentration-dependent discrimination of Ce4-treated samples, most pronounced for carrier-free, followed by KP-encapsulated Ce4 (Figure 9A). However, the strong impact of Cho on the outcome of the Pareto-scaled PLS analysis is in part also due to its high concentration in the cells relative to the other metabolites, as evidenced by the intense Cho resonance in the NMR spectrum (Figure 3). As opposed to unit variance scaling, Pareto scaling does not put equal weight onto each metabolite but only reduces the relative importance of dominant components, keeping the proportions of spectral peak intensities [76]. While clearly giving rise to less intense resonances, Cit has a strong impact with load values around 0.3 on the discrimination of cells treated mainly with PVP- but also KP-associated Ce4. For the same groups, a clear positive correlation also exists with Asp levels (load values around 0.2). Among the metabolites with moderate contributions to the separation (±0.1–±0.2) are PC and GPC with negative loadings mainly for Ce4-KP exposed samples. Moreover, decreased Cre levels correlate with Ce4 concentration, and Lac and Ala have negative load values for carrier-free Ce4 treated samples. On the other hand, higher levels of Suc (free Ce4, Ce4-KP), Fum, and Glu, as well as Lip (only for Ce4-PVP), correlate with increases in the drug dose.

Metabolic alterations within each subgroup (no carrier/KP/PVP) must be due to Ce4 concentration as the only independent variable. Differences between the subgroups must be related to Ce4-formulation. According to the PCA/PLS-DA (Figure 6 and Figure 7), the carrier material itself has only a small impact on the cell metabolic response. Therefore, different metabolite oPLS loadings between the three subgroups may be explained as follows: (i) the formulation results in an overall lower actual intracellular Ce4 concentration range, as shown in Figure 2; (ii) due to restrained drug release from the carriers, different concentration ranges of free Ce4 may be present in the cell samples of each subgroup; (iii) the carriers may account for different intracellular drug localization sites possibly resulting from altered uptake and transport pathways of Ce4 into and inside the cell. 

**Figure 10:**
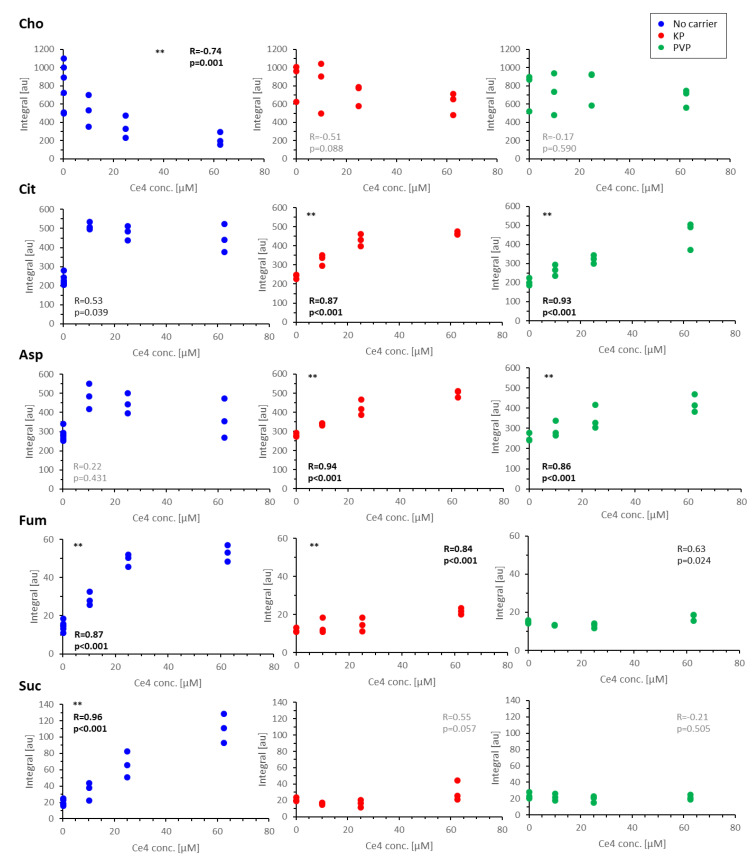
Plots of single metabolite integrals as a function of chlorin e4 (Ce4) concentration applied without carrier (blue), with Kolliphor P188 -micelles (KP, red), and with polyvinylpyrrolidone (PVP, green). Correlation analysis was performed by fitting with resulting R-values and the significance of correlation. Metabolites with highly significant (*p* < 0.001) Ce4-concentration-dependent levels are marked with **. Cho: choline; Cit: citrate; Asp: aspartate; Fum: fumarate; Suc: succinate.

For validation, single metabolites were evaluated by a targeted approach applying correlation analysis to the corresponding representative buckets. For this, multiple buckets belonging to the same metabolite were merged to yield one value for each assigned metabolite. Plots for selected metabolites exhibiting significant level changes are shown in Figure 10. From these plots, it becomes evident that the relative changes observed for Fum and Suc with a highly significant (*p* < 0.001) increase are comparable to the decrease of Cho in Ce4-exposed cells in the absence of carriers (Figure 10). The rise in Fum levels is less pronounced but still significant for cells treated with Ce4-KP. Cit and Asp levels almost doubled with high significance (*p* < 0.001) when cells were treated with Ce4 in the presence of KP or PVP. When treated with free Ce4, Cit, and Asp, levels jumped to the double value at the lowest Ce4 concentration and remained high with further Ce4 dose increase. In summary, 12 metabolites exhibited highly significant level changes with increasing Ce4 concentration without carriers, including decreased Cre, Tau, Urd, and Hxn and increased FA, sFA, Ac, Leu, and UTP (Figure S7). 

Exposure to Ce4-KP and Ce4-PVP only yielded four (increased Cit, Asp, Fum, and Glu, Figure S7) and two (Cit and Asp) highly significant metabolite level changes with Ce4 concentration, respectively. In the first case (no carrier), cell uptake of relatively high concentrations of free Ce4 seems to amount to a drug boost triggering a strong metabolic response, whereas the overall lower uptake and delayed release from PVP reveal the subtle response to lower concentrations of free Ce4. The eased drug release from the micellar environment (KP) consequently causes an intermediate response, which is in accordance with the data presented in Figure 6, Figure 7 and Figure 8. Finally, a simultaneous increase in Cit, Fum, and Suc was exceptionally observed for samples exposed to carrier-free Ce4 (Figure 9 and Figure 10). Since these metabolites are closely related to the tricarboxylic acid (TCA) cycle, the findings may point to an inhibitory primary or secondary toxic effect of free Ce4. The significant decrease in Cho levels may be related to a preferred membrane localization of free Ce4, as suggested by the data shown in Figure 5. With an easier release from micelles (KP) compared to PVP, a considerable amount of Ce4 may also be present in the membrane upon delivery with KP, explaining the slightly attenuated Cho decrease (*p* = 0.088, Figure 10) compared to carrier-free treatment.

#### 3.4.4. Discussion of Metabolic Changes

##### Components of the Phosphatidylcholine Metabolism

*Cho, FA, Cit, and Ac*

Cho, FA, Cit, and Ac are components involved in phosphatidylcholine metabolism. Cho decrease and increase in Cit, FA, sFA, and Ac, particularly at high intracellular Ce4 concentrations, indicate a perturbation of the pathways of FA and phospholipid synthesis or degradation [77]. Typically, the high cell proliferation of cancer cells is marked by elevated Cho and PC levels compared to healthy cells [78,79]. Consequently, the pronounced Cho decrease observed upon exposure to free Ce4 and Ce4-KP may indicate a downregulation of membrane synthesis and reduced cell proliferation. Such an effect has been reported for several antiproliferative drugs acting on the enzymatic activity involved in the biosynthesis of phospholipids [80,81]. However, in these studies, the Cho decrease is mostly accompanied by a PC decrease and GPC increase, which does not hold in the present work, so other mechanisms may be responsible. Reduced Cho levels may be related to the observed membrane localization shown in Figure 5. Both effects, PLC-upfield shift and Cho integral decrease, seem to correlate since they were each most pronounced with free Ce4 and least with PVP. Based on its high affinity for the Cho-containing head groups of phospholipids (Figure 5), a direct interaction of Ce4 that bears two carboxylate groups with free cationic Cho is conceivable but would require further investigation. The higher Cit levels may originate from the inhibited TCA cycle and consequently exported mitochondrial Cit to the cytoplasm, where it links to FA synthesis.

##### Components of the TCA Cycle

*Cit, Asp, Fum, and Suc*

Cit is a key compound of the TCA cycle and, in the cytoplasm, the link and starting point of FA synthesis. It is transported into the cytosol and cleaved, yielding acetyl-CoA and oxaloacetate (OAA) for further anabolic utilization, like FA synthesis from acetyl-CoA. Cytosolic Cit concentrations are typically low due to their rapid turnover [82]. The Ce4-concentration-dependent increase in Cit levels indicates an imbalance that can be caused by both enhanced Cit synthesis and enhanced mitochondrial release via the Cit carrier membrane protein [83] or reduced Cit consumption in the cytosol. OAA is, among others, also converted into Asp for nucleotide and polyamine synthesis. Since Cit and Asp levels were simultaneously increased in a Ce4-concentration-dependent manner, it is likely that the same pathway of stimulated nutrient production (FA, nucleotides) is related to the metabolic response observed in this study. 

Like Cit, both Suc and Fum are TCA cycle intermediates that are closely related, with Suc being directly converted to Fum via the enzyme succinate-dehydrogenase. While both metabolites are enhanced, the Suc increase is more pronounced (about six-fold at the highest Ce4 concentration) compared to three-fold for Fum (Figure 10). Thus, enhanced Fum levels can be a result of Suc overproduction or due to an inhibition of Suc and Fum degradation, respectively. This may be caused by an imbalance in the TCA cycle enzymatic conversions. On the other hand, the accumulation of Suc may also be a result of non-enzymatic formation via decarboxylation and oxidation of α-ketoglutarate as a response to oxidative stress, reducing the level of ROS [84,85]. However, since the experiments were performed under strict exclusion of light, ROS production primarily caused by Ce4 is rather unlikely.

##### Nucleobases, Nucleosides, Nucleotides, and Amines

Alterations in Cre and the pyrimidine/purine derivatives Urd, UTP, and Hxn reflect cellular adaptations in the nucleotide and energy metabolism in response to Ce4 exposure. It seems likely that the simultaneous Urd decrease and UTP increase are directly related to their enzymatic interconversion [86].

In summary, the metabolic alterations observed in HeLa cells exposed to different Ce4 concentrations can be interpreted as stimulating cellular defense mechanisms against the non-physiological porphyrinic invader (xenobiotic) to maintain the cellular metabolic state [87]. Since the cellular metabolic fingerprint obtained from the NMR spectrum represents a metabolic snapshot, the meaning of level changes in specific metabolites may be caused on either side by accumulation or consumption. Nevertheless, altered level changes and their magnitudes indicate which metabolic pathways are affected and are out of equilibrium. Notably, the polymeric carrier material, even though applied at a much higher concentration than the PS, had only little or no detectable effects on cell metabolism.

### 3.5. Limitations

While HR-MAS NMR has the potential to study a wide range of semi-solid materials with restricted mobility and short T_2_ relaxation times, larger structures entering a more solid-like phase are no longer observable by this technique. For example, most cellular proteins give rise to very broad lines, rendering them NMR-invisible under HR-MAS conditions. In the current study, the lack of PVP resonances in the cell HR-MAS NMR spectra indicates that PVP becomes part of a larger complex, possibly endosomal membranes, most likely leading to severe line broadening, thus causing its NMR invisibility. Nevertheless, the presence of intracellular PVP was indicated indirectly by its attenuating impact on Ce4 effects on the cell metabolite spectra.

Cell uptake pathways, intracellular fate, and metabolic responses of PS-carrier systems may turn out differently depending on the cell type [15]. Therefore, it will be of interest in future studies if the observed metabolic alterations described here for HeLa cells will be similarly transferable to other cell lines. Further, the application of serum-free incubation media, a commonly applied method in metabolomic cell intervention studies [88], excludes the impact of proteins during cell uptake. However, serum proteins may bind to the NP outer surface, possibly resulting in modifications of cell uptake and response. Finally, the complete exclusion of light may not be guaranteed at a 100% level, so subtle effects may be due to oxidative processes triggered by light. However, in the current experimental setup, dead cells were excluded, and the different incubation media were all applied simultaneously under the same conditions. In addition, metabolic antioxidant cell defense mechanisms in response to oxidative stress mainly involve alterations in the GSH/GSSG and NAD/NADH or NADP/NADPH levels [89], which was not observed in the current study.

## 4. Conclusions

In the current study, HR-MAS NMR was applied to cells exposed to different PS concentrations with and without two different carriers, namely block copolymer micelles and PVP, under the exclusion of light.

Addressing the intracellular fate of the components, the NMR data allow the following conclusions: (i) based on induced chemical shift changes of the cell-PLC resonance, the PS localizes in or near cellular membranes when applied in its free form and when associated with carriers. (ii) Once taken into the cells, the PS remains largely associated with PVP and is less tightly bound to the micelles. This was deduced from the observation that PS-induced effects were more strongly attenuated by PVP than by the KP-micelles. (iii) The micellar carrier material is cell-internalized, as was evidenced by the appearance of a PEG resonance in the cell spectra. (iv) The micellar components partly disassemble inside the cell since the PEG resonances arose from components with different diffusion properties. (iv) Intracellular PVP could only indirectly be traced via its attenuating impact on the PS-induced changes in cell metabolites. The current approach thus has offered the unique possibility to trace both the PS and the block copolymer inside the cell on a molecular and even atomic level. To our knowledge, this is the first time intracellular sub-molecular PS membrane localization has been detected via ring current-induced chemical shift perturbation of the phospholipid resonances. At the same time, examination of the small metabolite cell spectra and their multi- and univariate analyses indicated the metabolic response to the PS and carrier material under the exclusion of light.

Addressing the metabolic response of the cells, the NMR data allow the following conclusions: (i) the pure carrier material, PVP and KP-micelles, exhibits low toxicity based on the largely unchanged cell metabolic profile. (ii) The physiologic state of the cells was noticeably perturbed when exposed to the pure PS and correlated with the PS dose. (iii) The metabolic response to PS treatment was clearly attenuated when the PS was associated with KP-micelles and even more with PVP. (iv) Comparing PVP and KP-micelles, it can be delineated that, in general, carriers with a stronger binding capacity for the drug molecule are capable of reducing unwanted physiological perturbations more efficiently, thus maintaining cell homeostasis. This is an important aspect that may be extrapolated to other drugs that are likewise applied with comparable nano-platforms. In particular, for porphyrinic PSs, it is crucial to keep any activity or correspondence with their cellular environment as low as possible during the dark incubation and accumulation period before the selective phototoxic reaction is triggered by light irradiation. In this sense, the carrier not only fulfills the task of protecting the PS during its transport in the bloodstream and thus enhances PS accumulation in the diseased tissue but also protects the intracellular environment from the PS. Thus, potential side effects and dark toxicity can be further lowered. For the development of PS-based therapies, the presented data underline the importance of using carriers of physiologically inert material with high drug affinity to prevent premature PS release, reduce unwanted side effects, and thus improve patient compliance. Ideally, a PS remains inactive by its protecting carrier envelope in non-target tissue until cleared from the body. At the same time, photoactivity testing is crucial to obtain systems optimized for both dark conditions and upon light irradiation.

## Figures and Tables

**Table 1:** Cell sample groups and their labels used throughout this study (Ce4: chlorin e4, Ctrl: control, PVP: polyvinylpyrrolidone, KP: Kolliphor P188).

		Carrier	
Ce4 Conc. [μM]	None	PVP [208.3 μM]	KP [3 mM]
0	Ctrl1, Ctrl2	PVP	KP
10	Ce4-10	PVP-Ce4-10	KP-Ce4-10
25	Ce4-25	PVP-Ce4-25	KP-Ce4-25
62.5	Ce4-62.5	PVP-Ce4-62.5	KP-Ce4-62.5

**Table 2:** Signal assignment with chemical shift and multiplicity of proton resonances from HeLa-lysed cell suspension (PBS).

Compound	Abbreviation	Chemical Shift [ppm]	Multipl.	Group
*Amino acids*				
Alanine	Ala	1.477 3.779	(d) (q)	β-CH_3_ α-CH
Arginine	Arg	1.656 3.214	(m) (t)	γ-CH_2_ δ-CH_2_
Aspartate	Asp	2.689 2.814 3.894	(m) (m) (m)	β-CH_2_ β-CH_2_ α-CH
Glutamate	Glu	2.043 2.346 3.766	(m) (m) (t)	β-CH_2_ γ-CH_2_ α-CH
Glutamine	Gln	2.138 2.448 3.778	(m) (m) (t)	β-CH_2_ γ-CH_2_ α-CH
Glycine	Gly	3.558	(s)	α-CH_2_
Histidine ^a^	His	7.95 7.14	(s) (s)	2-CH (imid) 4-CH (imid)
Isoleucine	Ile	0.937 1.009 1.257 1.461 1.982 3.673	(t) (d) (m) (m) (m) (d)	δ-CH_3_ γ′-CH_3_ γ-CH_2_ γ-CH_2_ β-CH α-CH
Leucine	Leu	0.959 1.709 3.744	(d) (m) (t)	δ-CH_3_, δ′-CH_3_ γ-CH, β-CH_2_ α-CH
Lysine	Lys	1.462 1.703 1.883 3.003	(m) (m) (m) (t)	γ-CH_2_ δ-CH_2_ β-CH_2_ ε-CH_2_
Phenylalanine	Phe	7.317 7.367 7.411 3.994 3.273 3.131	(d) (t) (d) (m) (m) (m)	-CH -CH -CH α-CH β-CH_2_ β-CH_2_
Proline	Pro	2.03 2.352 3.333 3.415 4.145	(m) (m) (m) (m) (m)	γ-CH_2_ β-CH_2_ δ′-Ch_2_ δ″-CH_2_ α-CH
Threonine	Thr	1.33 3.587 4.257	(d) (d) (m)	γ-CH_3_ α-CH β-CH
Tyrosine	Tyr	6.887 7.182 3.943 3.205 3.052	(d) (d) (m) (m) (m)	-CH -CH α-CH β-CH_2_ β-CH_2_
Valine	Val	0.985 1.041 2.272 3.613	(d) (d) (m) (d)	γ-CH_3_ γ′-CH_3_ β-CH α-CH
*Peptides*				
Glutathione	GSH	2.166 2.554 2.958 3.780 3.787 4.577 8.393 8.587	(m) (m) (dd) (s) (t) (m)	β-CH_2_ (Glu) γ-CH_2_ (Glu) β′-CH_2_ (Cys) α″-CH (Gly) α-CH (Glu) α′-CH (Cys) -NH (Gly) -NH (Cys)
Lysine-containing peptide ^b^	LysP	1.462 1.703 3.003	(m) (m) (t)	γ-CH_2_ δ-CH_2_ ε-CH_2_
Tyrosine-containing peptide	TyrP	6.813 7.112	(br) (br)	-CH -CH
*Organic acids*				
Acetate	Ac	1.919	(s)	-CH_3_
Citrate	Cit	2.68 2.811	(d) (d)	-CH_2_ -CH_2_
Formic acid	For	8.45	(s)	-CH
Fumarate	Fum	6.519	(s)	CH=CH
Lactate	Lac	1.33 4.121	(d) (q)	β-CH_3_ α-CH
Succinate	Suc	2.405	(s)	-CH_2_
*Amines*				
β-Alanine	β-Ala	2.57 3.18	(t) (t)	α-CH_2_ β-CH_2_
Creatine	Cre	3.03 3.934	(s) (s)	-CH_3_ -CH_2_
Taurine	Tau	3.264 3.421	(t) (t)	SO_3_H-CH_2_ NH_2_-CH_2_
*Choline-containing compounds*				
Choline	Cho	3.208 3.528 4.067	(s) (m) (m)	-N^+^(CH_3_)_3_ N-CH_2_ O-CH_2_
Glycerophosphocholine	GPC	3.233 3.695 4.338	(s, br) (m) (m)	-N^+^(CH_3_)_3_ N-CH_2_ O-CH_2_
Phosphocholine	PC	3.224 3.605 4.166	(s) (m) (m)	-N^+^(CH_3_)_3_ N-CH_2_ O-CH_2_
Phosphatidylcholine ^b^	PLC	3.273	(s, br)	-N^+^(CH_3_)_3_
*Nucleobases, nucleosides, and nucleotides*				
Adenosine phosphate ^c,d^	AXP	8.62 8.228	(s) (s)	8-CH 2-CH
Hypoxanthine	Hxn	8.188 8.208	(s) (s)	8-CH 2-CH
Inosine/Adenosine ^d^	Ino/Ado	8.235 8.358 6.101 4.781 4.471	(s) (s) (d) (t) (m)	8-CH 2-CH 1′-CH (Rib) 2′-CH (Rib) 3′-CH (Rib)
1-Methylnicotinamide ^e^	1-MNA	9.283 8.982 8.897 4.48	(s) (s) (d) (s)	2-CH 6-CH 4-CH N^+^-CH_3_
NAD^+^	NAD^+^	9.325 9.117 8.804 8.43 8.166 8.139 6.074	(s) (d) (d) (s) (t) (s) (d)	2-CH (Nic) 6-CH (Nic) 4-CH (Nic) 2′-CH (Ade) 5-CH (Nic) 8′-CH (Ade) 1-CH (Rib)
NADP^+^	NADP^+^	9.305	(s)	2-CH (Nic)
Nicotinamide ^e^	NA	8.933 8.706 8.235 7.582	(s) (d) (d) (dd)	2-CH 6-CH 4-CH 5-CH
Uridine monophosphate ^c^	UMP	8.14 5.998	(d) (d)	6-CH (Ura) 5-CH (Ura)
Uracil	Ura	7.534 5.797	(d) (d)	6-CH 5-CH
Uridine	Urd	7.897 5.894 5.922 4.357 4.229 4.123 3.828	(d) (d) (d) (m)	6-CH (Ura) 5-CH (Ura) 1′-CH (Rib) 2′,3′-CH (Rib) 4′-CH (Rib) 5a′-CH_2_ (Rib) 5b′-CH_2_ (Rib)
Uridine triphosphate	UTP	7.965 5.967 5.985 4.375 4.282	(d) (d) (d) (m)	6-CH (Ura) 5-CH (Ura) 1′-CH (Rib) 2′,3′-CH (Rib) 4′-CH (Rib)
*Nucleotide-/phosphate-sugars*				
Ribose-5-Phosphate	R5P	5.388 5.257 4.129	(dd) (d)	1-CH 1-CH 2-CH
UDP-N-Acetyl-glucosamine	UNGlc	5.515 2.079 4.378 4.289 5.963 5.985 7.969	(dd) (s) (d) (d) (d)	1-CH (Glc) Ac-CH_3_ 2′,3′-CH (Rib) 4′-CH (Rib) 5-CH (Ura) 1′-CH (Rib) 6-CH (Ura)
UDP-N-Acetyl-galactosamine	UNGal	5.548 2.085 4.378 4.289 5.963 5.985	(dd) (s) (d) (d)	1-CH (Gal) Ac-CH_3_ 2′,3′-CH (Rib) 4′-CH (Rib) 5-CH (Ura) 1′-CH (Rib)
UDP- Glucose/UDP- Glucuronic acid ^d^	UGlc(A)	5.612	(dd)	1-CH (Glc(A))
*Lipids*				
Fatty acids	FA	0.867 1.192 1.302	(br) (br) (br)	ω-CH_3_ (-CH_2_)_n_ (-CH_2_)_n_
Fatty acids (saturated)	sFA	0.883 1.615	(t) (br)	ω-CH_3_ β-CH_2_-
Lipids ^b^	Lip	0.936 1.293 1.628 2.073 2.292	(t) (br) (br) (br) (br)	ω-CH_2_- (-CH_2_)_n_ β-CH_2_ -CH_2_-CH= α-CH_2_
*Others*				
Ethanol	Eth	1.193 3.668	(t) (q)	-CH_3_ -CH_2_
Polyethylene glycol ^f^	PEG	3.711	(s)	-CH_2_
Polypropylene glycol ^f^	PPG	1.16	(br)	-CH_3_

^a^ slight chemical shift variation (pH-sensitive); ^b^ only or mainly visible in diffusion-weighted spectra (Figure S2); ^c^ only in cell samples treated with Ce4; ^d^ assignment ambiguous; ^e^ low concentration (mainly visible in the summed spectrum); ^f^ only in cell samples treated with KP.

## Data Availability

The data presented in this study are available on request from the corresponding authors.

## Supplementary Materials

The following are available online at https://www.mdpi.com/article/10.3390/pharmaceutics15092324/s1, Figure S1: HR-MAS ^1^H^1^H-TOCSY spectra of lysed HeLa cell suspension in PBS with resonance assignments, Figure S2: HR-MAS ^1^H-1D PROJECT, ^1^H-1D NOESY, and ^1^H-1D DOSY spectra of lysed HeLa cell suspension in PBS with resonance assignments of the 1D DOSY spectra, Figure S3: DOSY bi-exponential fitting curves, Figure S4: HR-MAS ^1^H-T_2_-edited and ^1^H-diffusion-edited spectra of 30 mM PVP in PBS, Figure S5: HR-MAS ^1^H-diffusion-edited spectra of HeLa cells incubated with 30 mM PVP, with 30 mM KP and reference spectra of pure PVP and KP (30 mM in PBS), Figure S6: PCA scores plot displayed in Figure 6 with labeling of samples according to the three independently prepared cell batches (a, b, c) and loading plot for the principal components PC 1 and PC 2, Figure S7: Plots of single metabolite integrals as function of Ce4 concentration applied without a carrier, with KP-micelles, and with PVP. Table S1: PLS-DA model statistics for individual sample classes.

## Abbreviations

BCM, block copolymer micelles; Ce4, chlorin e4; Ce6, chlorin e6; cmc, critical micelle concentration; DMEM, Dulbecco’s Modified Eagle’s Medium; DOSY, diffusion-ordered spectroscopy; EPR, enhanced permeability and retention; FCS, fetal calf serum; HR-MAS, high-resolution magic angle spinning; HSA, human serum albumin; KP, Kolliphor P188; NOESY, nuclear Overhauser enhancement spectroscopy; NP, nanoparticle; OAA, oxaloacetate; oPLS, orthogonal partial least squares; P, penicillin; PBS, phosphate-buffered saline; PCA, principal component analysis; PDT, photodynamic therapy; PEG, polyethylene glycol; PES, polyethersulfone; PLS-DA, partial least squares discriminant analysis; PPG, polypropylene glycol; PQN, probabilistic quotient normalization; PROJECT, periodic refocusing of J evolution by coherence transfer; PS, photosensitizer; PVP, polyvinylpyrrolidone; RMSEC, root mean square error of calibration; RMSECV, root mean square error of cross validation; ROS, reactive oxygen species; S, streptomycin; T/E, trypsin-EDTA; TCA, tricarboxylic acid; TOCSY, total correlation spectroscopy. For metabolite abbreviations, see Table 2.
