# Supplementary material for: Intracellular Fate of the Photosensitizer Chlorin e4 with Different Carriers and Induced Metabolic Changes Studied by 1H NMR Spectroscopy
Source: Pharmaceutics. 2023 Sep 15;15(9):2324. doi: 10.3390/pharmaceutics15092324 (PMC10537485; doi:10.3390/pharmaceutics15092324)
Supplement: Supplementary file 1 [file pharmaceutics-15-02324-s001.zip › pharmaceutics-2599964-supplementary.pdf]

Supplementary Materials

Intracellular Fate of the Photosensitizer Chlorin e4 with Different Carriers and Induced Metabolic Changes Studied by ^{1}H NMR Spectroscopy

Martina Vermathen [1,*], Tobias Kämpfer [1,2], Jean-Marc Nuoffer [3,4] and Peter Vermathen [2,5,6,*]

1 Department of Chemistry, Biochemistry and Pharmaceutical Sciences, University of Bern, 3012 Bern, Switzerland; tobias.kaempfer@unibe.ch
2 Department of BioMedical Research, University of Bern, 3008 Bern, Switzerland
3 Institute of Clinical Chemistry, Bern University Hospital, 3010 Bern, Switzerland; jean-marc.nuoffer@insel.ch
4 Department of Pediatric Endocrinology, Diabetology and Metabolism, University Children's Hospital of Bern, 3010 Bern, Switzerland
5 University Institute of Diagnostic and Interventional Neuroradiology, Bern University Hospital, University of Bern, 3010 Bern, Switzerland
6 Translational Imaging Center (TIC), Swiss Institute for Translational and Entrepreneurial Medicine, 3010 Bern, Switzerland
* Correspondence: martina.vermathen@unibe.ch (M.V.); peter.vermathen@insel.ch (P.V.); Tel.: +41-(0)31-684-3948 (M.V.); +41-(0)31-632-3559 (P.V.)

Content

Figure S1 A-C: HR-MAS ^{1}H^{1}H-TOCSY spectrum of lysed HeLa cell suspension in PBS.

Figure S2 A, B: HR-MAS ^{1}H-1D PROJECT, ^{1}H-1D NOESY, and ^{1}H-1D DOSY spectra of lysed HeLa cell suspension in PBS.

Figure S3: DOSY bi-exponential fitting curves of data plots peak intensity versus gradient strength for single peaks.

Figure S4: HR-MAS ^{1}H-T2- and ^{1}H-diffusion-edited spectra of 30 mM PVP in PBS.

Figure S5: HR-MAS ^{1}H-diffusion-edited spectra of HeLa-cells incubated with 30 mM PVP, with 30 mM KP, and reference spectra of pure PVP and KP (30 mM in PBS).

Figure S6A, B: PCA scores plot displayed in Figure 6 with labelling of samples according to the three independently prepared cell batches (a, b, c). Loading plot for the principal components PC 1 and PC 2 of the scores plot.

Figure S7: Plots of single metabolite integrals as function of Ce4 concentration applied without carrier, with KP-micelles, and with PVP.

Table S1: PLS-DA model statistics for individual sample classes, number of LVs: 2.

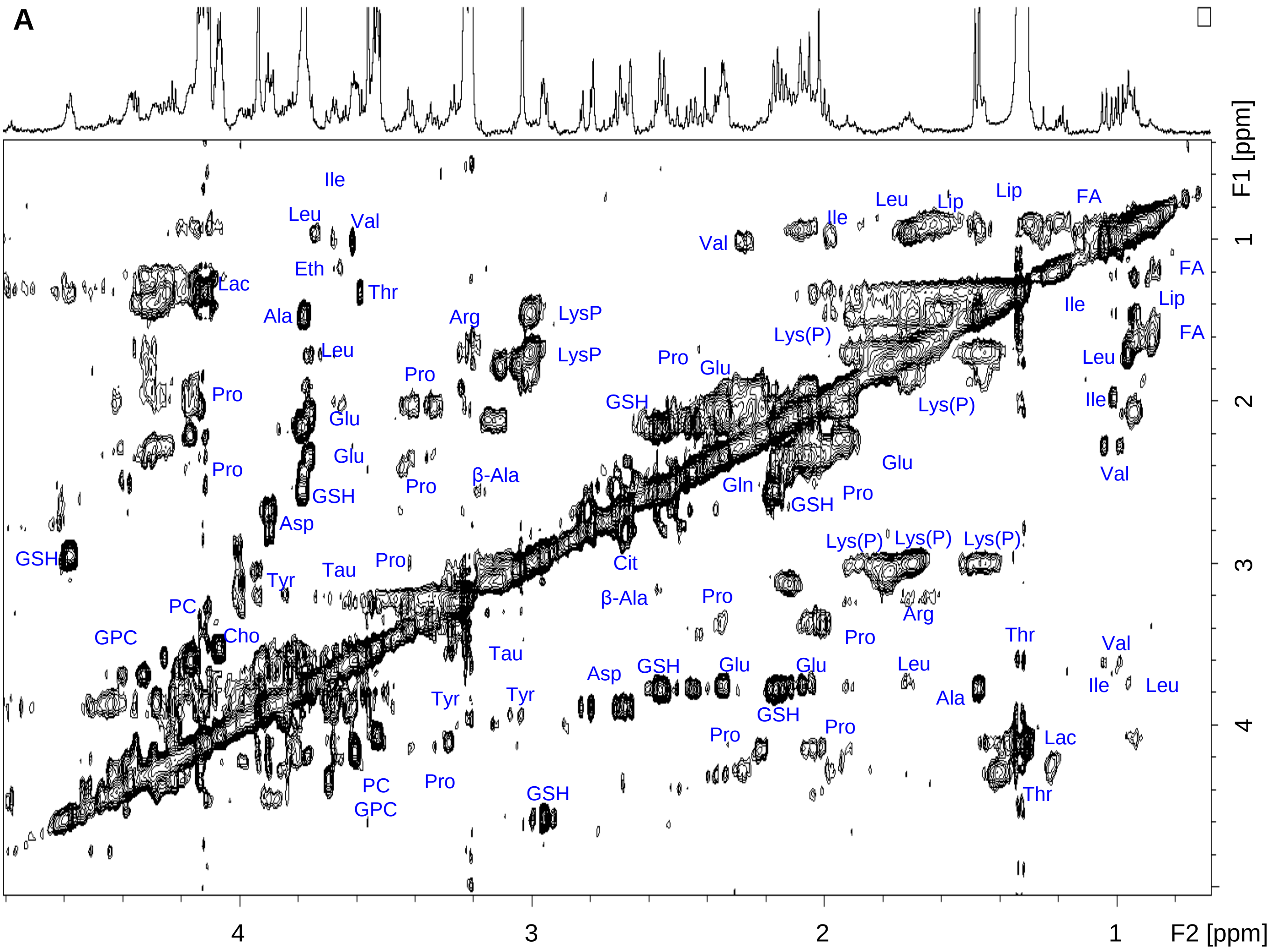

Figure S1 A: HR-MAS $^{1}H^{1}H$-TOCSY spectrum (aliphatic region) of lysed HeLa cell suspension in PBS with resonance assignments. Ala: Alanine; β-Ala: β-Alanine; Arg: Arginine; Asp: Aspartate; Cho: Choline; Cit: Citrate; Eth: Ethanol; FA: Fatty acids; Gln: Glutamine; Glu: Glutamate; GPC: Glycerophosphocholine; GSH: Glutathione; Ile: Isoleucine; Lac: Lactate; Leu: Leucine; Lip: Lipids; Lys: Lysine; LysP: Lysine containing peptide; PC: Phosphocholine; Pro: Proline; Tau: Taurine; Thr: Threonine; Tyr: Tyrosine; Val: Valine.

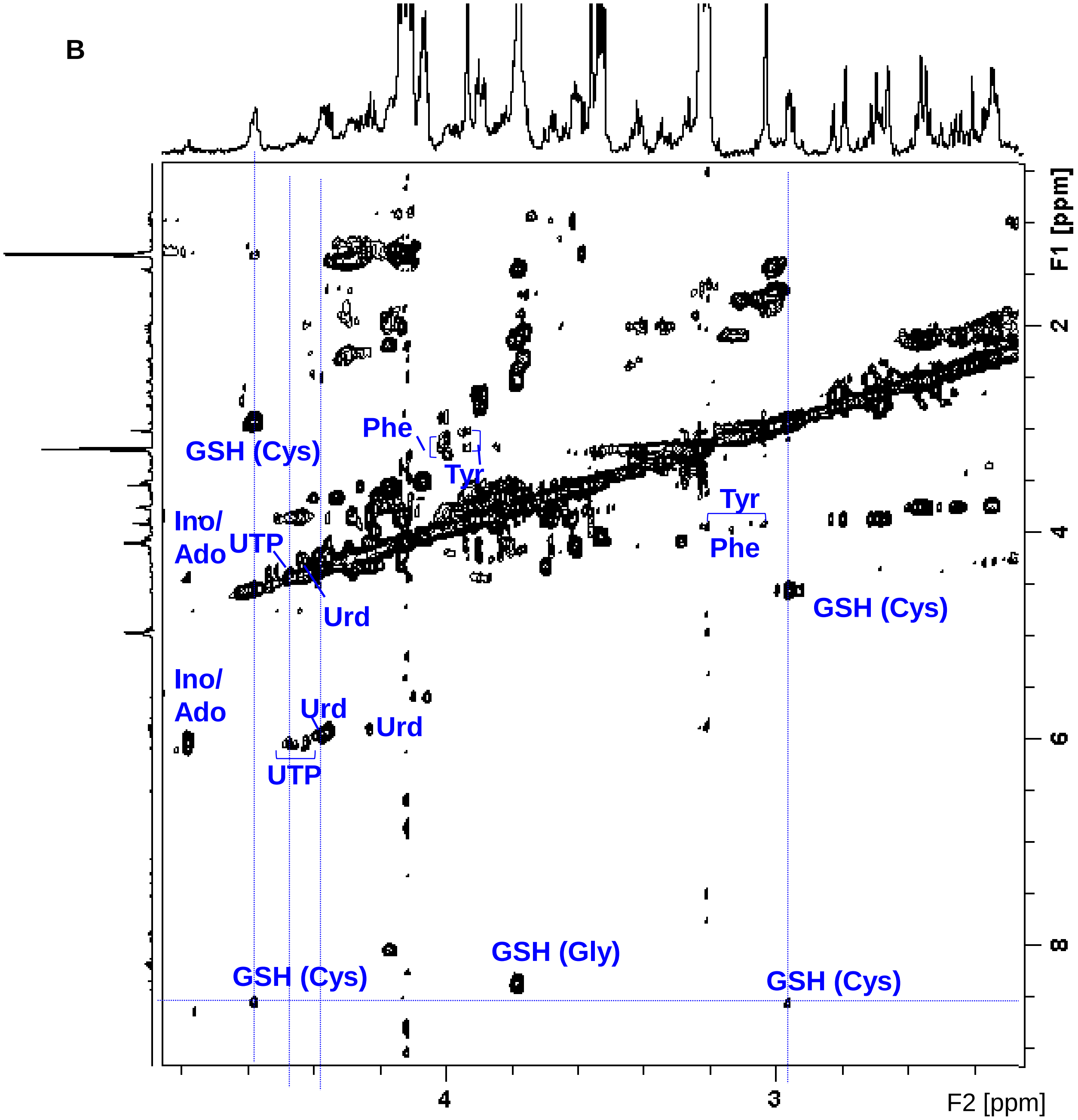

Figure S1 B: HR-MAS $^{1}H^{1}H$-TOCSY spectrum (2.2 - 4.8 ppm (F2) x 0.5 – 9 ppm (F1)) of lysed HeLa cell suspension in PBS with resonance assignments. Cys: Cysteine; GSH: Glutathione; Gly: Glycine; Ino/Ado: Inosine/Adenosine; Phe: Phenylalanine; Tyr: Tyrosine; Urd: Uridine; UTP: Uridine triphosphate.

C

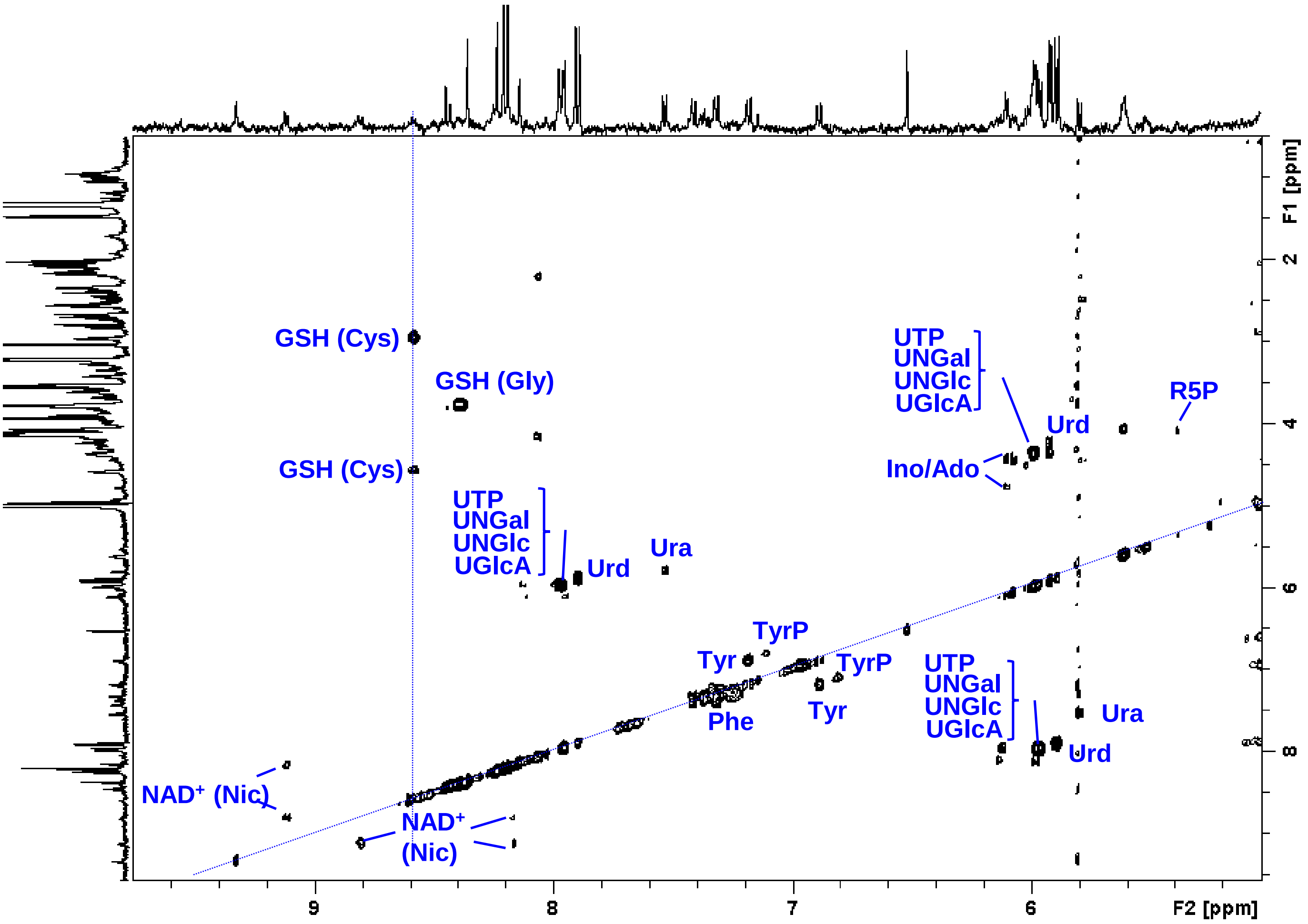

Figure S1 C: HR-MAS ^{1}H^{1}H-TOCSY spectrum (aromatic region) of lysed HeLa cell suspension in PBS with resonance assignments. Cys: Cysteine; GSH: Glutathione; Gly: Glycine; Ino/Ado: Inosine/Adenosine; Nic: Nicotinamide; Phe: Phenylalanine; R5P: Ribose-5-PhosphateTyr: Tyrosine; TyrP: Tyrosine containing peptide; UGlcA: UDP-Glucuronic acid; UNGal: UDP-N-Acetyl-galactosamine; UNGlc: UDP-N-Acetyl-glucosamine; Ura: Uracil; Urd: Uridine; UTP: Uridine triphosphate.

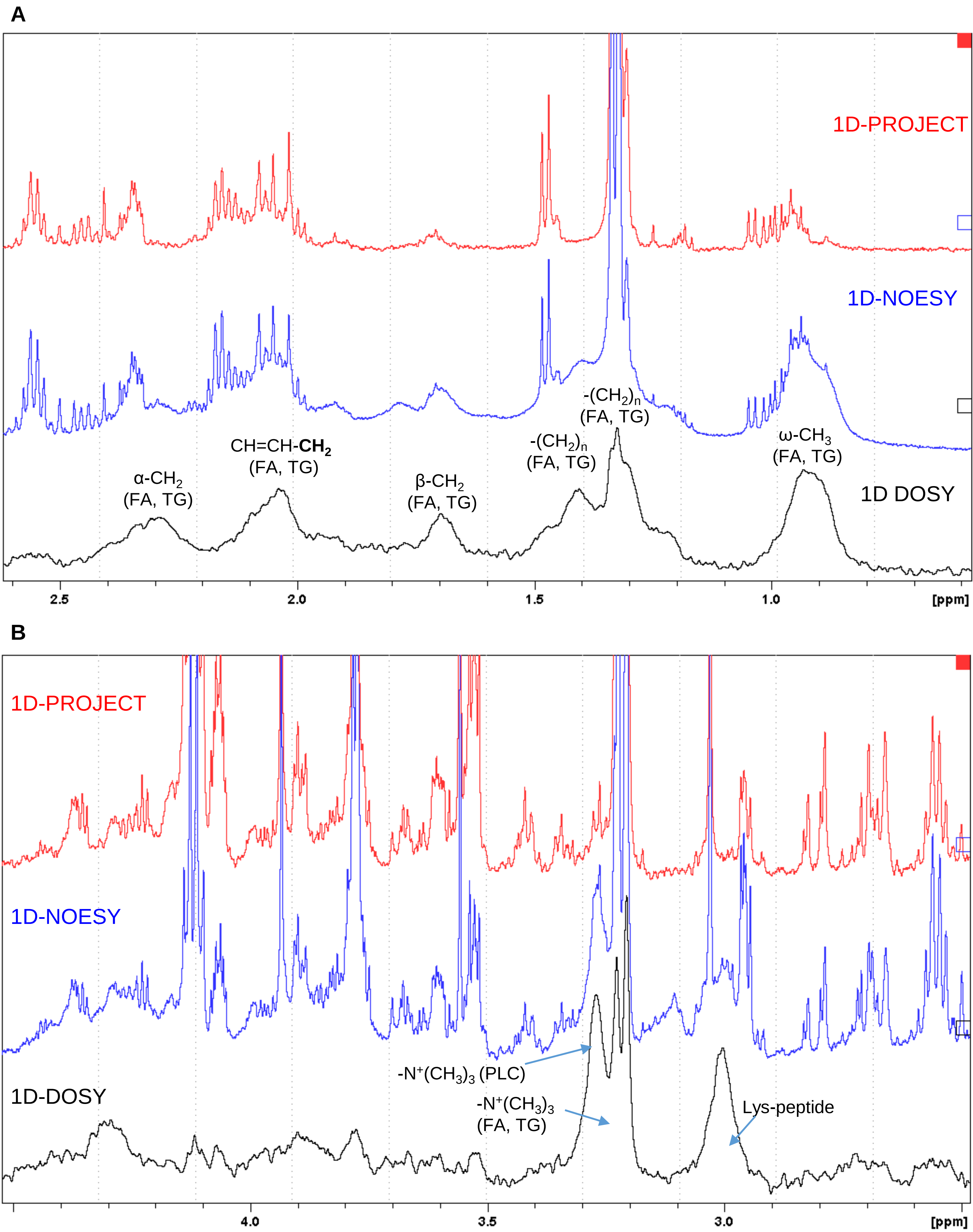

Figure S2: HR-MAS ^{1}H-1D PROJECT (red), ^{1}H-1D NOESY (blue), and ^{1}H-1D DOSY (black) spectra of lysed HeLa cell suspension in PBS with resonance assignments of the 1D DOSY spectra. (**A**) Spectral region 0 – 2.6 ppm, (**B**) spectral region 2.5 – 4.5 ppm. FA: Fatty acids; TG: Tri-Glycerides; PLC: Phosphatidylcholine.

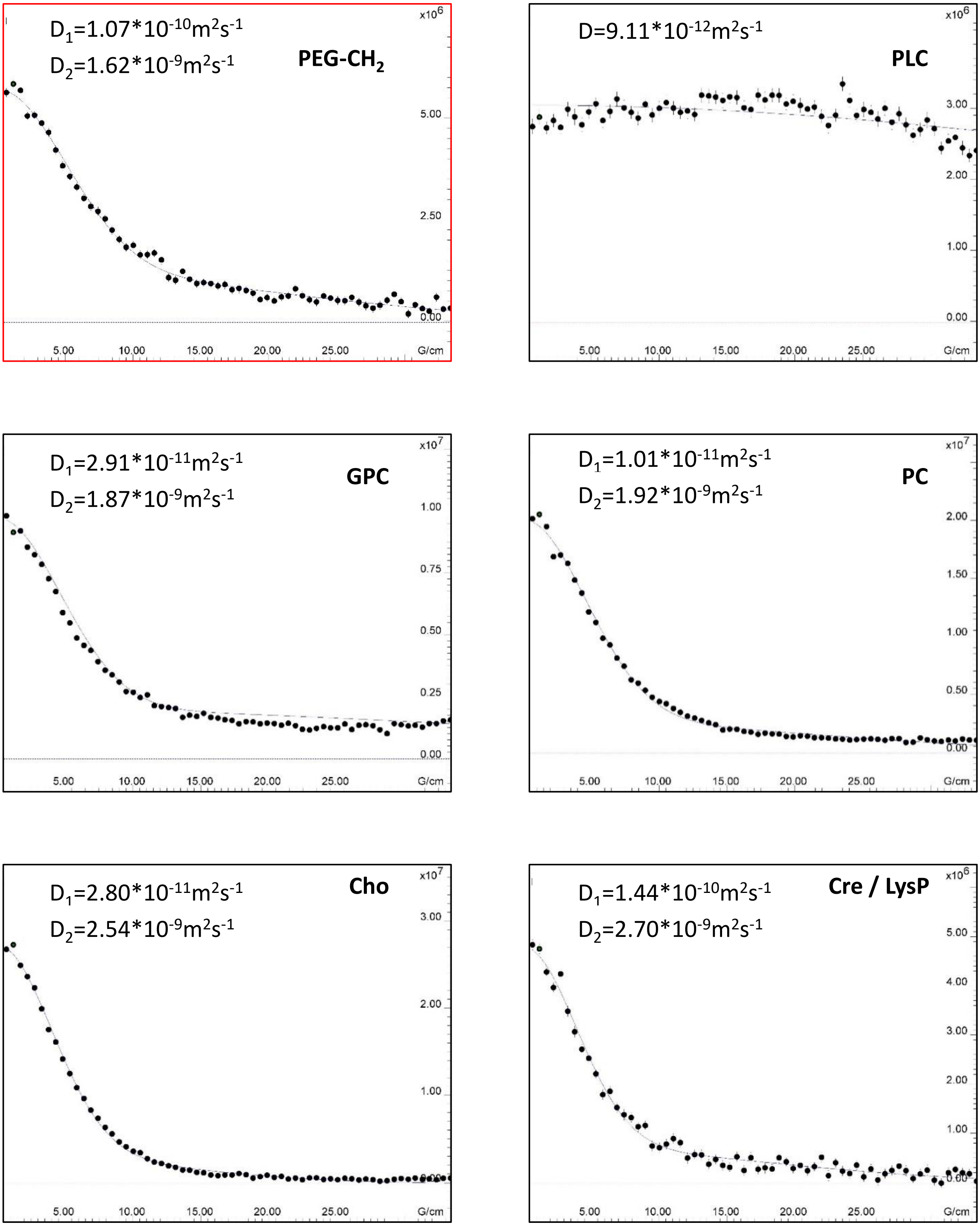

Figure S3: DOSY bi-exponential fitting curves of data plots peak intensity versus gradient strength for single peaks. PEG: Polyethylene glycol; PLC: Phosphatidylcholine; GPC: Glycerophosphocholine; PC: Phosphocholine; Cho: Choline; Cre: Creatine; LysP: Lysine containing peptide.

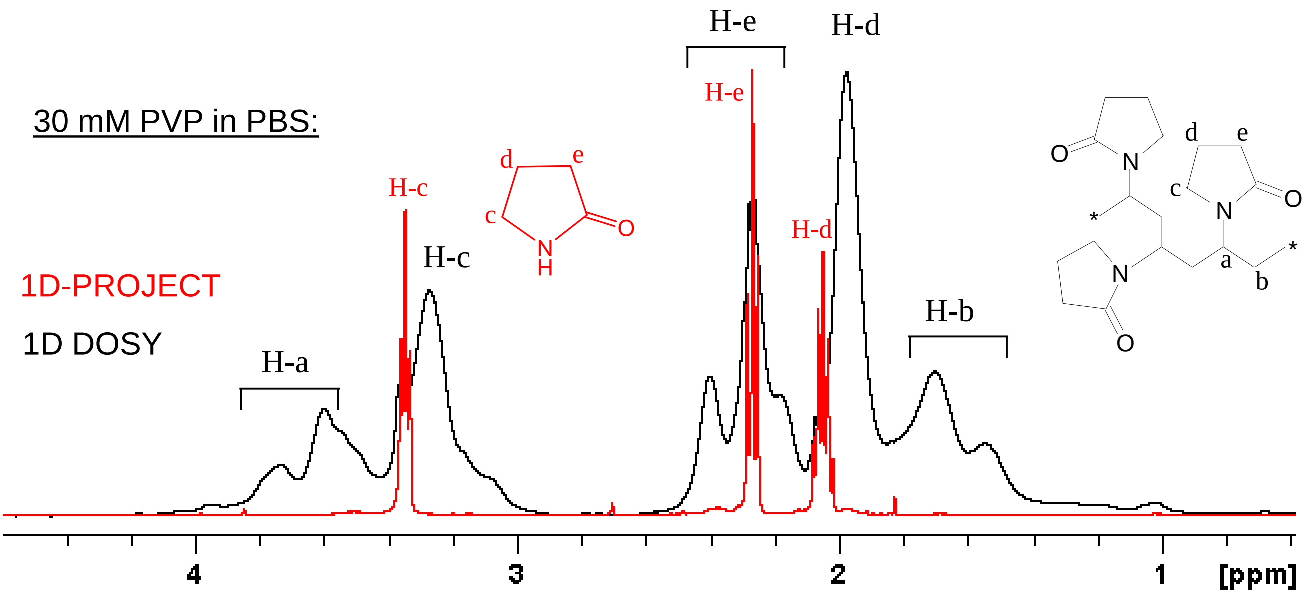

Figure S4: HR-MAS ^{1}H-T_2-edited (red) and ^{1}H-diffusion-edited (black) spectra of 30 mM polyvinylpyrrolidone (PVP) in PBS. The spectra demonstrate that PVP resonances are suppressed upon application of the T_2-filter applied in the cell spectra and only the resonances of the low MW impurity (pyrrolidone, red structure) remain, whereas the PVP (black structure) resonances remain visible upon application of the diffusion filter used in the cell spectra.

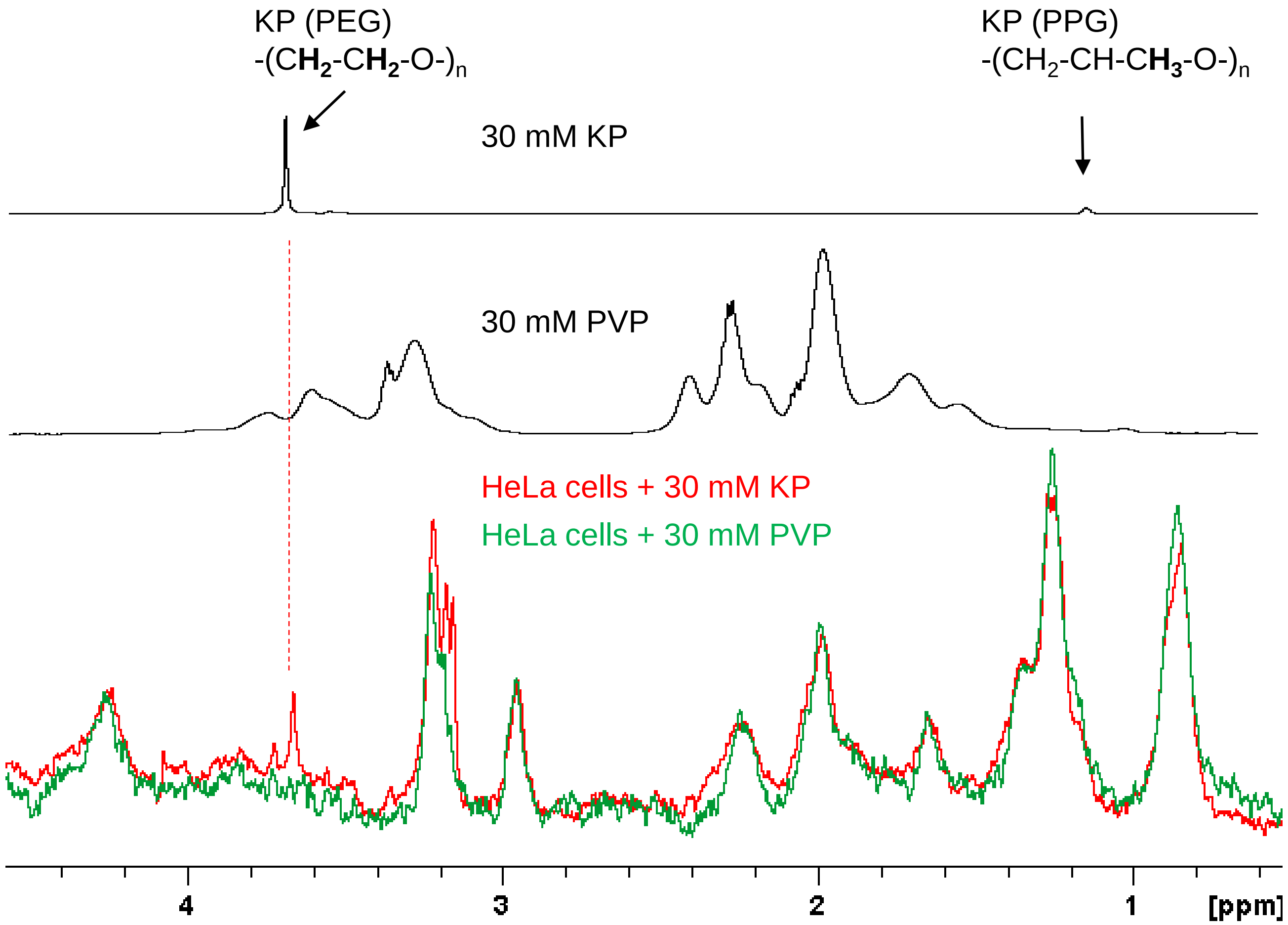

Figure S5: HR-MAS ^{1}H-diffusion-edited spectra of HeLa-cells incubated with 30 mM polyvinylpyrrolidone (PVP, green), with 30 mM Kolliphor P188 (KP, red) and reference spectra of pure PVP and KP (30 mM in PBS, black). Whereas the KP polyethylene glycol (PEG) resonance appears in the cell spectra (red line), the PVP resonances remain invisible in the cell spectra.

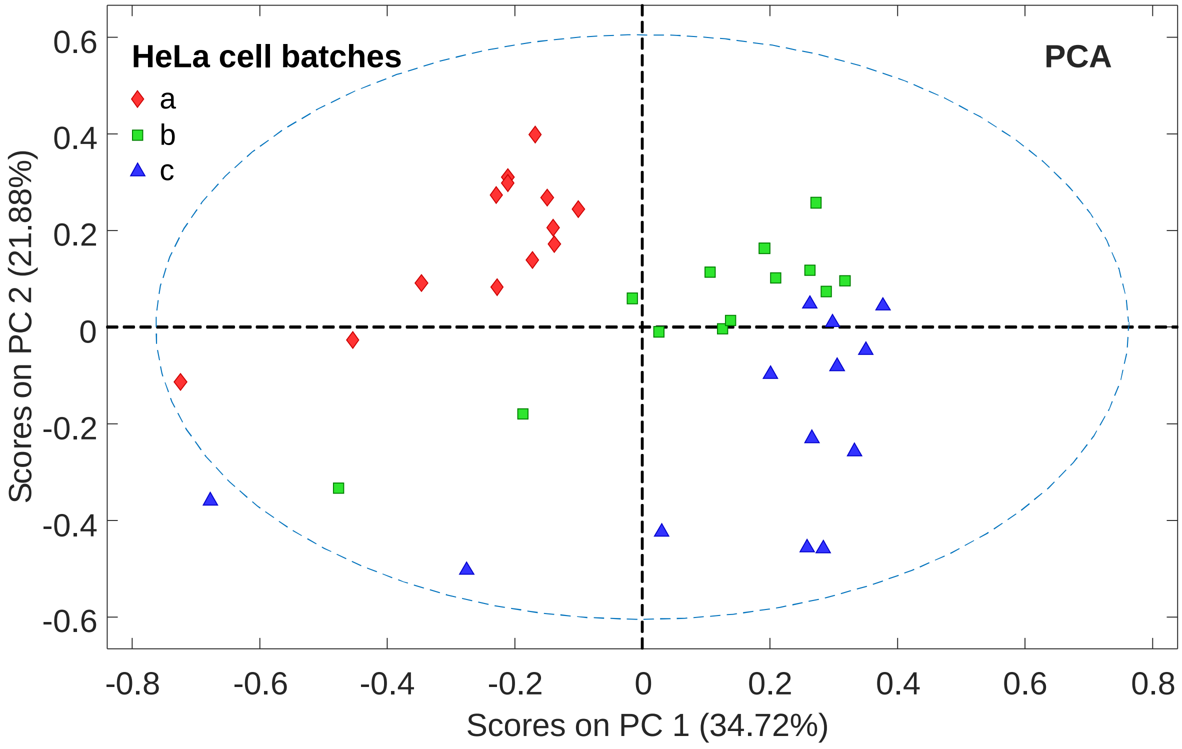

Figure S6 A: PCA scores plot displayed in Figure 6 with labelling of samples according to the three independently prepared cell batches (a, b, c). A separation of the batches is seen mainly Along PC-2. Dotted ellipsoid represents 95% confidence level.

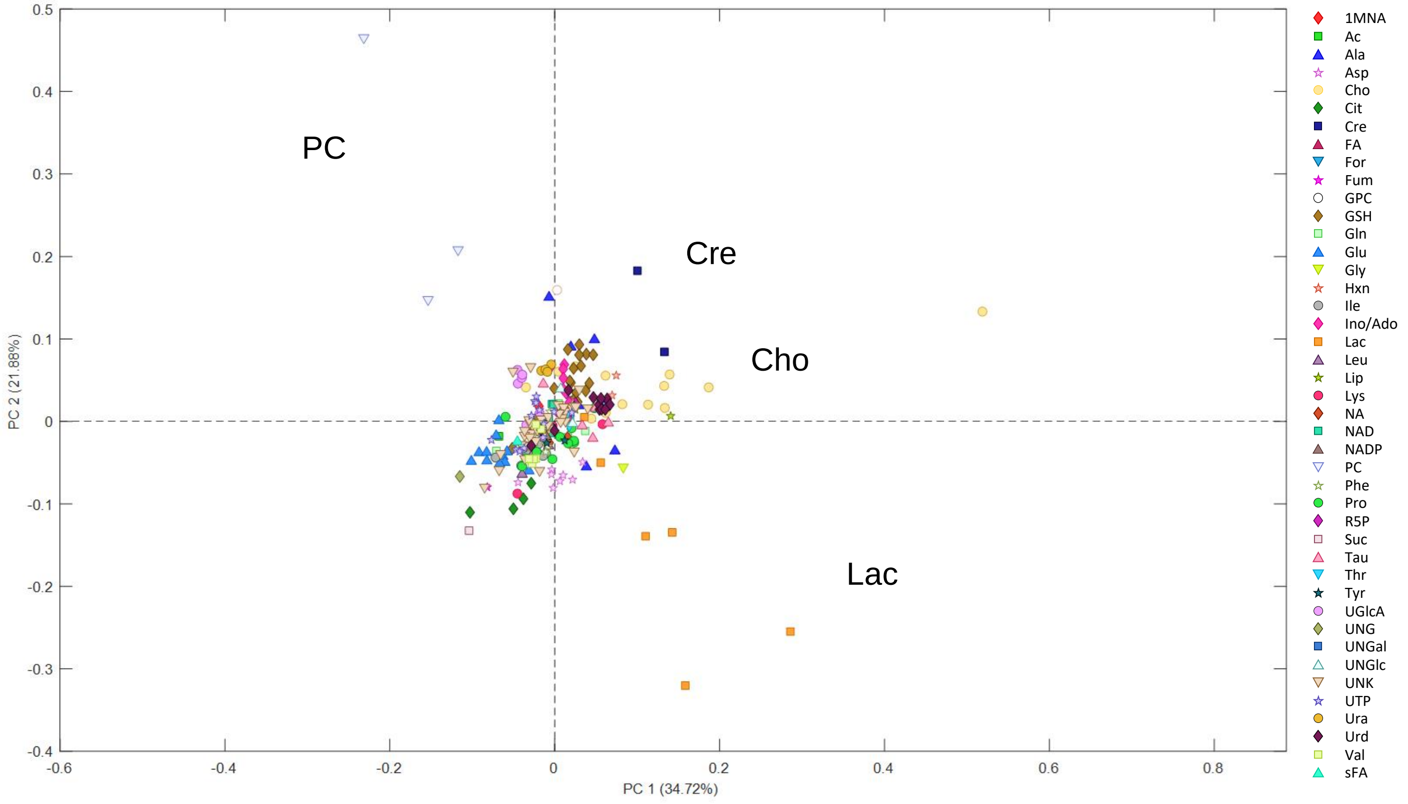

Figure S6 B: Loading plot for the principal components PC 1 and PC 2 of the scores plot displayed in Figure S6 A. Main contributors to separation of the different batches are PC and Lac. 1-MNA: 1-Methylnicotinamide; Ac: Acetate; Ala: Alanine; Asp: Aspartate; Cho: Choline; Cit: Citrate; Cre: Creatine; FA: Fatty acids; For: Formic acid; Fum: Fumarate; GPC: Glycerophosphocholine; GSH: Glutathione; Gln: Glutamine; Glu: Glutamate; Gly: Glycine; Hxn: Hypoxanthine; Ile: Isoleucine; Ino/Ado: Inosine/Adenosine; Lac: Lactate; Leu: Leucine; Lip: Lipids; Lys: Lysine; NA: Nicotinamide; PC: Phosphocholine; Phe: Phenylalanine; Pro: Proline; R5P: Ri-bose-5-Phosphate; Suc: Succinate; Tau: Taurine; Thr: Threonine; Tyr: Tyrosine; UGlcA: UDP-Glucose/UDP-Glucuronic acid; UNG: UNGlc/UNGal; UNGal: UDP-N-Acetyl-galactosamine; UNGlc: UDP-N-Acetyl-glucosamine; UNK: Un-known; UTP: Uridine triphosphate; Ura: Uracil; Urd: Uridine; Val: Valine; sFA: Fatty acids (saturated).

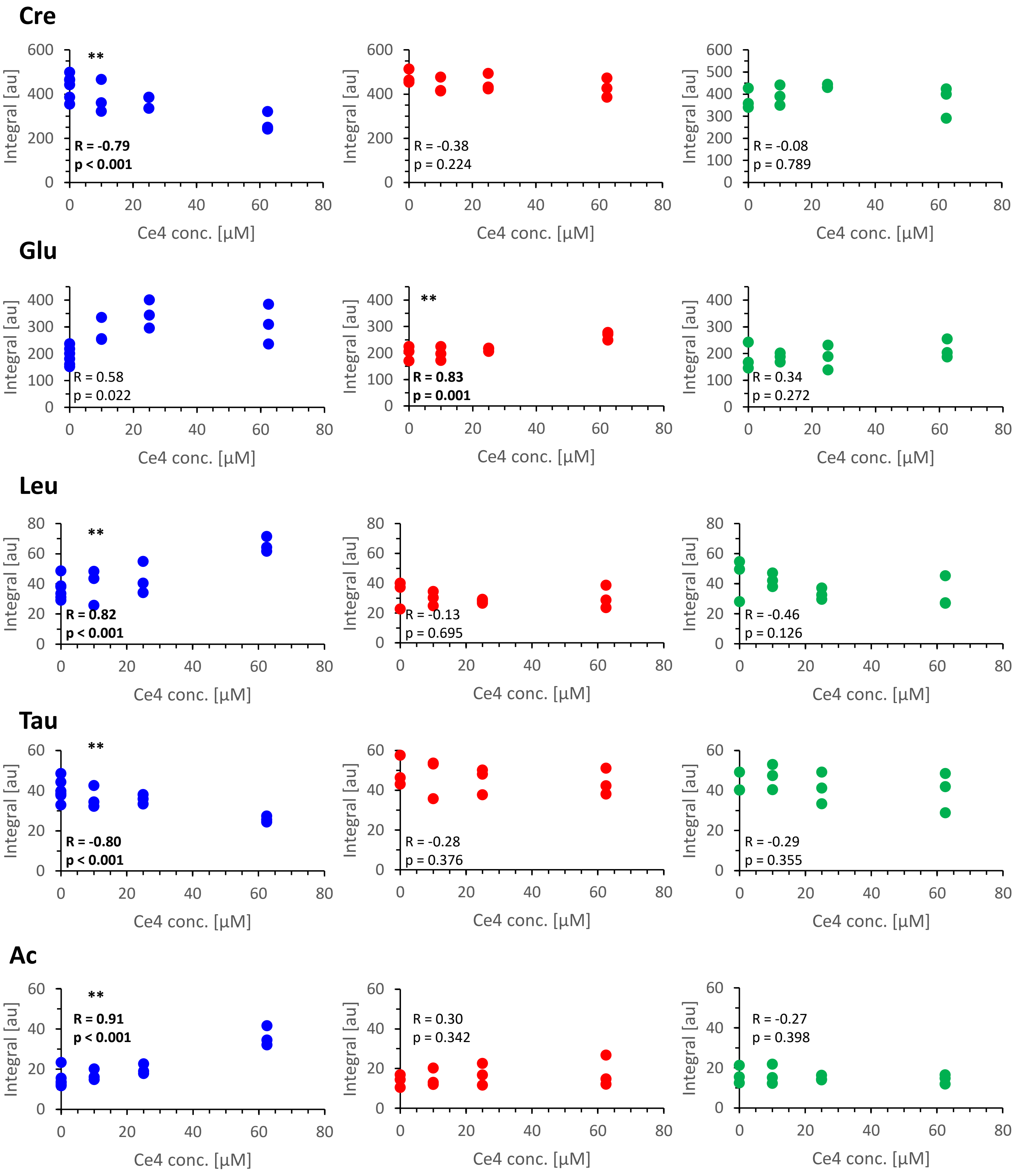

Figure S7: Plots of single metabolite integrals as function of Chlorin e4 (Ce4) concentration applied without carrier (blue), with Kolliphor P188-micelles (KP, red), and with polyvinylpyrrolidone (PVP, green). Correlation analysis performed by fitting with resulting R-values and significance of correlation. Metabolites with highly significant ($p < 0.001$) Ce4-concentration dependent levels are marked with **. Cre: Creatine; Glu: Glutamate; Leu: Leucine; Tau: Taurine; Ac: Acetate.

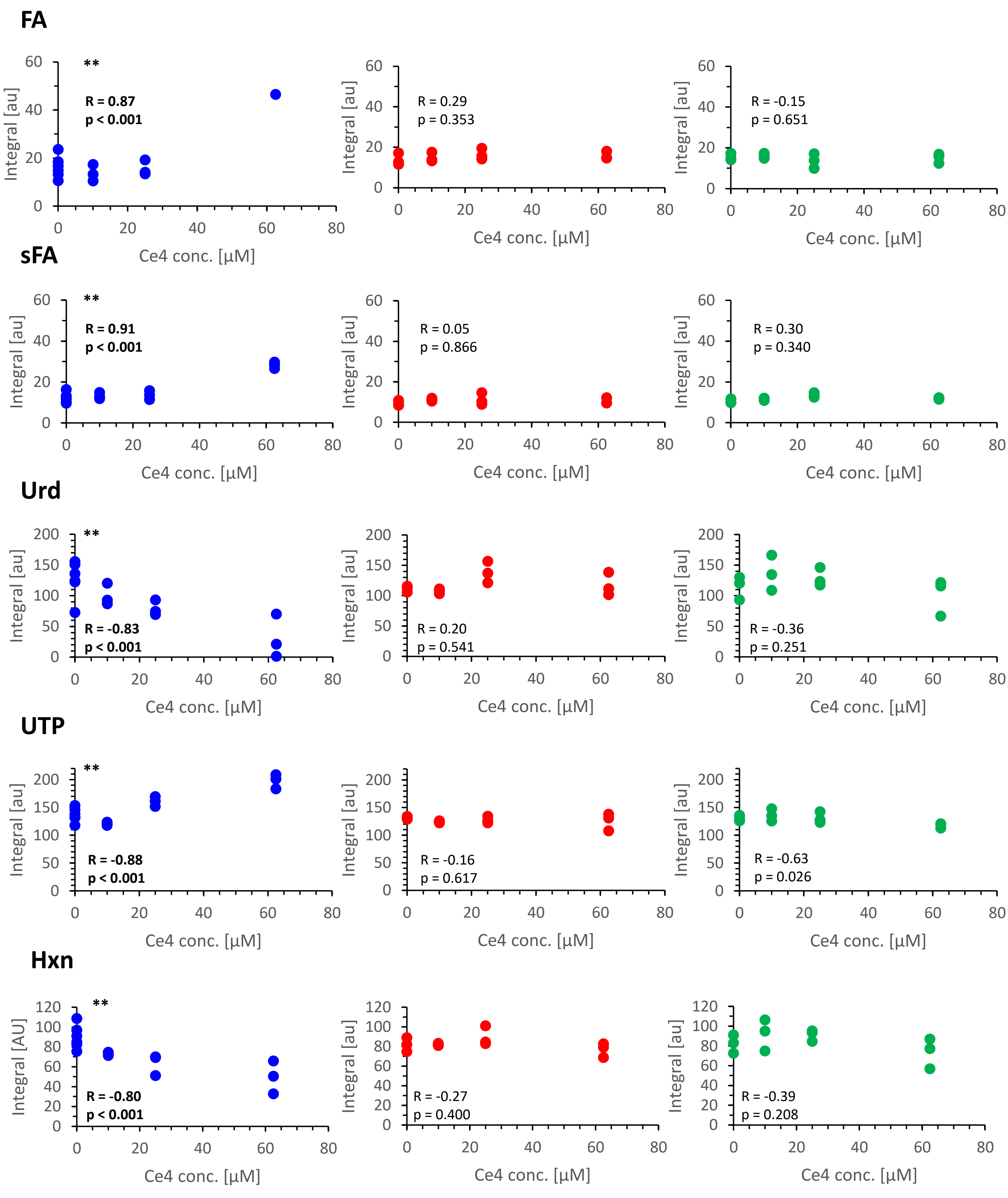

Figure S7 (continued): Plots of single metabolite integrals as function of Chlorin e4 (Ce4) concentration applied without carrier (blue), with Kolliphor P188-micelles (KP, red), and with polyvinylpyrrolidone (PVP, green). Correlation analysis performed by fitting with resulting R-values and significance of correlation. Metabolites with highly significant ($p < 0.001$) Ce4-concentration dependent levels are marked with **. FA: Fatty acids; sFA: Fatty acids (saturated); Urd: Uridine; UTP: Uridine triphosphate; Hxn: Hypoxanthine.

Table S1: PLS-DA model statistics for individual sample classes, number of LVs: 2. Ce4: Chlorin e4; Ctrl: Control; KP: Kolliphor P188; PVP: Polyvinylpyrrolidone.

Model class	Classification error		R^2	
	Calculated	Cross validated	Calculated	Cross validated
Ce4-10	0.06	0.22	0.22	0.05
Ce4-25	0.097	0.07	0.19	0.06
Ce4-62.5	0	0	0.67	0.51
Ctrl 1, 2	0.17	0.40	0.19	0.09
KP	0.21	0.35	0.08	0.005
KP-Ce4-10	0.24	0.44	0.04	0
KP-Ce4-25	0.39	0.40	0.04	0.002
KP-Ce4-62.5	0.11	0.28	0.17	0.07
PVP	0.18	0.33	0.09	0.019
PVP-Ce4-10	0.21	0.54	0.06	0.002
PVP-Ce4-25	0.28	0.47	0.03	0.005
PVP-Ce4-62.5	0.15	0.32	0.09	0.018
